# Towns and trails drive carnivore movement behaviour, resource selection, and connectivity

**DOI:** 10.1186/s40462-022-00318-5

**Published:** 2022-04-08

**Authors:** Jesse Whittington, Mark Hebblewhite, Robin W. Baron, Adam T. Ford, John Paczkowski

**Affiliations:** 1 Park Canada, Banff National Park Resource Conservation, PO Box 900, Banff, AB T1L 1K2 Canada; 2 Wildlife Biology Program, Department of Ecosystem and Conservation Sciences, W.A. Franke College of Forestry and Conservation, University of Montana, 32 Campus Drive, Missoula, MT 59801 USA; 3 Department of Biology, Faculty of Science, University of British Columbia, Kelowna, BC V1V 1V7 Canada; 4 Alberta Environment and Parks, Kananaskis Region, 201, 800 Railway Avenue, Canmore, AB T1W 1P1 Canada

**Keywords:** Connectivity, Conservation, Corridor, Hidden Markov, Movement ecology, Human development, Resource selection, Step selection, Utilization distribution

## Abstract

**Background:**

Global increases in human activity threaten connectivity of animal habitat and populations. Protection and restoration of wildlife habitat and movement corridors require robust models to forecast the effects of human activity on movement behaviour, resource selection, and connectivity. Recent research suggests that animal resource selection and responses to human activity depend on their behavioural movement state, with increased tolerance for human activity in fast states of movement. Yet, few studies have incorporated state-dependent movement behaviour into analyses of Merriam connectivity, that is individual-based metrics of connectivity that incorporate landscape structure and movement behaviour.

**Methods:**

We assessed the cumulative effects of anthropogenic development on multiple movement processes including movement behaviour, resource selection, and Merriam connectivity. We simulated movement paths using hidden Markov movement models and step selection functions to estimate habitat use and connectivity for three landscape scenarios: reference conditions with no anthropogenic development, current conditions, and future conditions with a simulated expansion of towns and recreational trails. Our analysis used 20 years of grizzly bear (*Ursus arctos*) and gray wolf (*Canis lupus*) movement data collected in and around Banff National Park, Canada.

**Results:**

Carnivores increased their speed of travel near towns and areas of high trail and road density, presumably to avoid encounters with people. They exhibited stronger avoidance of anthropogenic development when foraging and resting compared to travelling and during the day compared to night. Wolves exhibited stronger avoidance of anthropogenic development than grizzly bears. Current development reduced the amount of high-quality habitat between two mountain towns by more than 35%. Habitat degradation constrained movement routes around towns and was most pronounced for foraging and resting behaviour. Current anthropogenic development reduced connectivity from reference conditions an average of 85%. Habitat quality and connectivity further declined under a future development scenario.

**Conclusions:**

Our results highlight the cumulative effects of anthropogenic development on carnivore movement behaviour, habitat use, and connectivity. Our strong behaviour-specific responses to human activity suggest that conservation initiatives should consider how proposed developments and restoration actions would affect where animals travel and how they use the landscape.

**Supplementary Information:**

The online version contains supplementary material available at 10.1186/s40462-022-00318-5.

## Background

Global increases in human activity threaten wildlife populations and as a result, many conservation programs have increased their focus on ecological connectivity [1].

Connectivity analyses of animal movement are used to identify dispersal routes between populations [2, 3], seasonal migrations routes [e.g. 4, and to highlight natural and anthropogenic pinch points to movement (i.e. wildlife corridors) as priority areas for conservation [5, 6]. There are, however, many ways to measure or evaluate connectivity [7]. Fahrig et al. [7] succinctly describe how landscape structure and movement behaviour can be used to differentiate Noss and Merriam connectivity. Most studies focus on Noss connectivity, which is defined as ‘the extent to which patches are connected to one another by similar habitat or corridors’ [8]. Noss connectivity identifies movement routes and corridors based on landscape structure (e.g. habitat quality or resistance to movement), but lacks metrics of individual movement success such as predicted rates of movement between habitat patches or through wildlife corridors [7]. Merriam connectivity, on the other hand, is defined as ‘the degree to which a landscape facilitates or impedes movement of organisms among resource patches’ [9]. Importantly, Merriam connectivity estimates individual movement rates between patches. The rapid growth of animal movement and step selection models has increased opportunities for simulating realistic animal movements in order to predict habitat use and to estimate Merriam connectivity [10, 11].

Animals typically intersperse fast movements with strong directional persistence (e.g. exploratory and travelling behaviour) with slow movements that have low directional persistence [e.g. encamped feeding and resting behaviour, 12. The interplay between fast and slow movements occurs as animals move between and within habitat patches [13] and also when animals leave secure habitat to navigate matrix habitat that may contain unsuitable forage, high risk of disturbance, or high risk of mortality [14–16]. Multiple studies have found strong state-dependent responses to anthropogenic development [15, 17–19] and that ignoring movement behaviour can generate poor estimates of connectivity [20].

Movement models and step selection analyses offer complementary approaches for understanding how human activity affects movement behaviour, resource selection, and Merriam connectivity. Hidden Markov movement models are commonly used to identify latent behavioural states of movement from animal location data [21, 22]. Transition probabilities between behavioural states can also be modelled as a function of spatial and temporal covariates. For example, Creel et al. [19] used a three-state model for African wild dogs (*Lycaon pictus*) and found that the probability of transitioning to a fast state of movement increased outside of protected areas, perhaps because of lower prey availability, and decreased in areas near human activity. Most hidden Markov movement models do not, however, address animal selection or avoidance of resources, which is important when trying to predict where animals will travel on the landscape.

Step selection analyses are a subset of spatial point-process models that are increasingly used to estimate relative selection of resources [23] and then to predict spatial variation in the intensity of habitat use [24]. Several studies have incorporated step selection functions (SSFs) into Noss connectivity analyses by first creating spatial predictions of habitat use and then transforming predictions into resistance layers for cost-distance or circuit theory analyses [2, 6, 25]. Other studies have used the derived resistance surfaces to simulate animal movements [26–28]. Only one study, to our knowledge, estimated Merriam connectivity by first simulating movements directly from the SSF and then estimating individual-based movement rates between populations [10].

Simulated individual-based paths are appealing because they can incorporate sequential, probabilistic movement decisions related to landscape features, speed of travel, and directional persistence [29]. Integrated step selection analyses interact spatial covariates with step lengths and turn angles as continuous variables [29, 30], but cannot be used clearly differentiate state specific responses to anthropogenic development. Predicting movement state probabilities from hidden Markov models and then incorporating predicted state probabilities into step selection models and subsequent simulations could provide insights into the effects of human activity on movement behaviour, spatial predictions of habitat use for foraging-resting versus hunting-travelling, and individual-based estimates of connectivity.

Quantifying the effects of current and future anthropogenic development on movement behaviour, resource selection, habitat patches, and connectivity may support better land use decision making for wildlife conservation and management of ecosystem-level processes [31, 32]. Here, we focused on the movements of two large carnivores in a transboundary region of Banff National Park (BNP), AB, Canada where transportation infrastructure, outdoor recreation, and urban areas occupy much of the prime habitat in the valley bottoms of the mountainous landscape. We focus on the movements of grizzly bears (*Ursus arctos*) and wolves (*Canis lupus*) because of their management relevance, threatened status, and important ecological roles [e.g. 33, 34. We used 20 years of grizzly bear and wolf telemetry data to develop seasonal, hidden Markov models and state-dependent SSFs. We used the movement models and SSFs to simulate animal paths under three landscape scenarios, from which we assessed changes in habitat quality and Merriam connectivity. Based on grizzly bear and wolf responses to human activity in other studies [30, 34–36], we expected grizzly bears and wolves to select linear features (e.g., road, trails) as efficient travel routes when travelling, while avoiding areas near towns and areas with high trail and road density, especially in their slow movement states. Finally, we expected that high quality habitat for slow and fast states around towns would decrease from current to future conditions due to an expanded town footprint and increased recreational trail density [37]. We also expected that the expanded anthropogenic development would reduce Merriam connectivity, which we measured as the proportion of simulated paths that travelled around the mountain towns of Banff and Canmore, AB. Building on the growing field of movement ecology, we provide a flexible approach to generate movement-based estimates of habitat quality and connectivity that can be applied to other taxa and systems.

## Methods

### Study area

The study area encompassed 17,450 km^2^ of the Canadian Rockies within and adjacent to BNP (51.2° N, 115.5° W, Additional file 1: Figure S1). We defined the extent of the study area based on movements of radio-collared wolves and grizzly bears monitored from 2000 to 2020. The study area contained rugged topography, short summers and long cold winters. See Whittington et al. [38] for a description of vegetation and the predator–prey community.

The study area contained the tourist towns of Banff and Canmore and several hamlets that occupied the centre of the Bow Valley. Linear features such as the Trans Canada highway, a national railway, and secondary roads bisected the study area. Like many global protected areas [39], human activity within the study area increased steadily over the last 20 years [40], with the potential for increasing impacts on wildlife connectivity [37]. BNP currently receives over 4 million visitors per year, mostly concentrated in summer. Most anthropogenic developments and recreational activities were concentrated near roads within the Bow Valley. Backcountry areas in the northeastern portion of the study area received minimal human use.

### Telemetry data

Researchers fit wolves and grizzly bears with Global Positioning System (GPS) collars to collect data from 2000 to 2020. Researchers captured and collared grizzly bears using a combination of culvert traps and free-range darting and wolves using a net shot from a helicopter under University and Federal government capture and Animal Care permits. Researchers programmed collars to collect GPS locations every two hours. GPS collars had high fix rates with low habitat-induced fix-rate bias [41]. We obtained a large sample of locations from both front and backcountry areas (Fig. 2, Additional file 1: Figures S1–S3).

### Statistical analyses

**Fig. 1:**
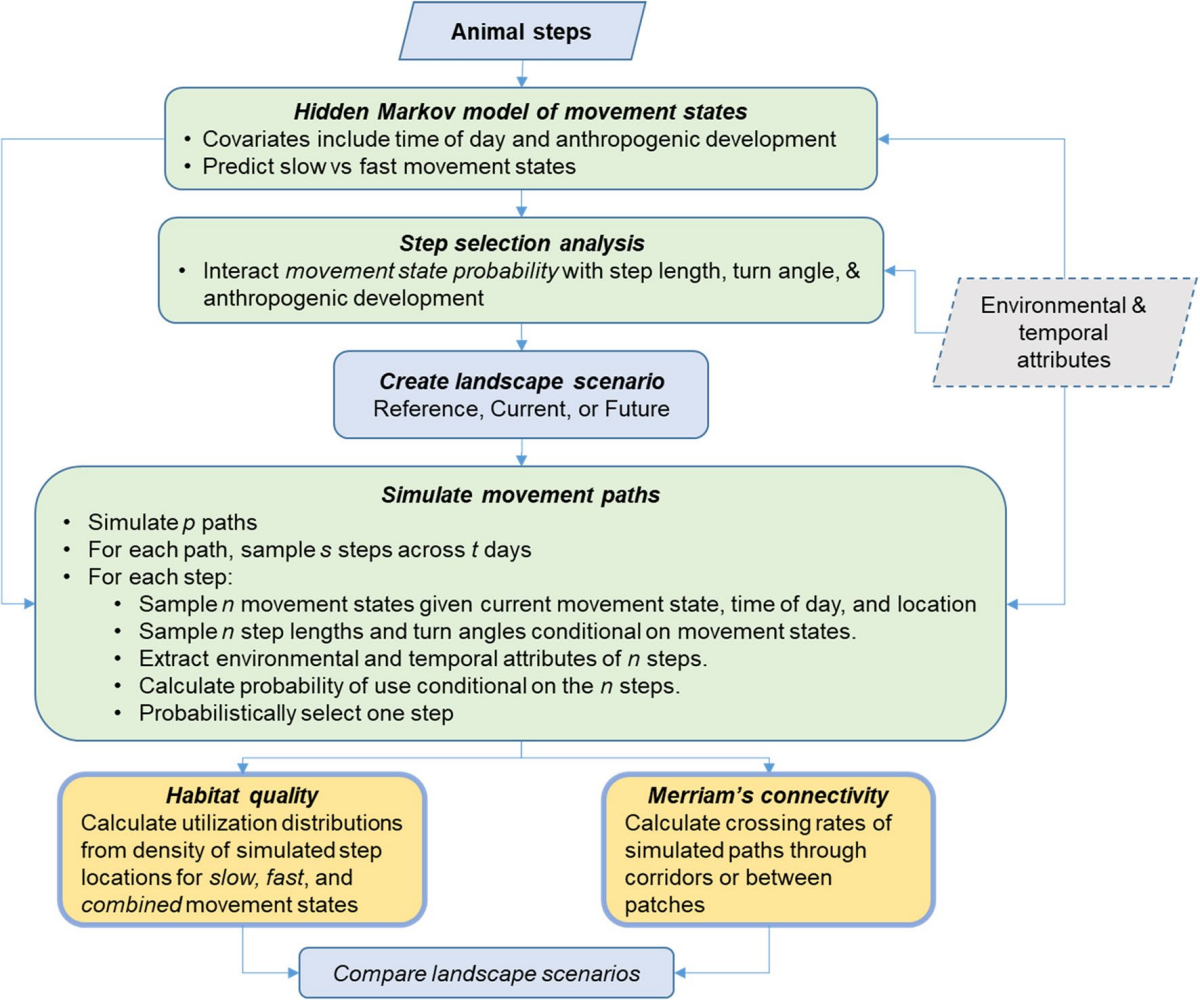
Conceptual and methodological model used to assess the effects of anthropogenic disturbance on movement behaviour, resource selection, habitat quality, and connectivity. We first used hidden Markov models to estimate the effects of anthropogenic development on movement behaviour and to classify data into slow and fast states of movement. Second, we used those state probabilities in SSF models to evaluate state-specific responses to anthropogenic development. Next, we used the combination of hidden Markov movement models and SSF models to simulate movement paths under multiple landscape scenarios. We used simulated locations to estimate habitat use (utilization distributions) for slow, fast, and all steps combined. We used simulated paths to estimate Merriam's connectivity through movement corridors. We compared results from current and future conditions to a reference condition with no anthropogenic development

We used a three stage, individual-based modeling approach to quantify carnivore responses to anthropogenic features and connectivity (Fig. 1). Here, we provide an overview of our methods and then later provide additional details for each step of the analysis. First, we applied hidden Markov models to animal movement data to both assess the effects of anthropogenic features and time of day on behavioural states of movement and to classify the data into slow and fast movement states. Second, we integrated movement states into SSFs, such that each SSF contained interactions between movement state, directional persistence, and speed of travel. We also included interactions between movement states and anthropogenic features. We used results of the SSF to assess state-specific responses to anthropogenic features. Third, we used the combination of hidden Markov models and SSFs with covariates to simulate realistic individual-based movements. We simulated movement paths under three landscape conditions reflecting reference, current, and future levels of anthropogenic development. Reference represented a null model of potential habitat with no anthropogenic development. For each landscape scenario and state, we calculated habitat use (utilization distributions) from which we could assess changes to the spatial distribution and amount of high-quality habitat for slow, fast, and combined movement states. Finally, we examined how current and proposed human activity affected through-transect connectivity, as a summary estimate of Merriam connectivity, near Banff and Canmore. We provide data and R scripts to fit the hidden Markov movement models, fit the step selection functions, and simulate paths in Additional file 3.

#### Movement state

We fit hidden Markov models to grizzly bear and wolf GPS step lengths and turn angles so that we could incorporate movement behaviour into SSFs and to create biologically realistic simulations of animal movement. We used functions from the *moveHMM* package version 1.7 to fit hidden Markov models [21]. For each species and season, we fit two-state movement models to reflect slow (feed and rest behaviour) and fast (travelling and hunting) movements, following previous studies of GPS movement [12]. We note that interpretation of movement behaviours for slow and fast states can differ between wolves and grizzly bears. Wolves are obligate predators that feed for hours to days at predation sites and thus are likely foraging, resting, or denning in slow states, and hunting/traveling in fast states. Grizzly bears are omnivores and can thus forage on berries, roots, and herbaceous plants either in concentrated habitat patches (slow movements) or while travelling (fast states). Regardless, we associate resting behaviour with slow states for both species. We fit movement models with the gamma distribution for step length and the circular von Mises distribution for turn angles [29]. We included time of day (cosine of hour) as a covariate to allow for diurnal variation in the frequency of slow and fast states. We also included proximity to town and trail-road density calculated using a 500 m radius (km/km^2^) as we expected carnivores to transition to fast states when near areas with high human activity. We evaluated five exponential and linear decay functions for proximity to town with asymptotes between 500 m and 5 km (Additional file 2, Table S1). For each GPS location, we predicted the probability of being in a fast state (*p*_*Fast*_), which we then incorporated into the SSF below (Fig. 1). We used parameters from the resulting movement models to simulate movement states, step lengths, and turn angles in path simulations below.

#### Step selection

We developed grizzly bear and wolf SSFs to first assess how anthropogenic development affected seasonal wolf and grizzly bear movements (Fig. 1) and then to simulate movements across three land use scenarios. We fit a full SSF model using conditional logistic regression with strata for paired used and available locations [29]. We sampled availability with using the pooled distribution of step lengths and turning angles so that we assessed resource selection relative to all available habitat within a two-hour step window. We generated 20 random steps for each observed step. We used generalized estimating equations (GEE) with clusters for individual animals to remove biases in variance associated with temporal autocorrelation of GPS locations [42]. The full model contained 13 topographic and habitat based covariates that have influenced grizzly bear and wolf resource selection in other studies [36, 43, 44]. Anthropogenic covariates included proximity to town with an exponential decay, trail-road density (linear and quadratic terms), on–off trails, on–off roads, and on–off the railway. We added interactions between anthropogenic covariates, probability of fast state, and time of day. We included parameters for step length and turn angle plus interactions between fast state probability *p*_*Fast*_ and step length, turn angle, and time of day (cosine of hour). Thus, the full model included the following covariates: land cover classification, normalized difference vegetation index, fractional snow cover, aspect, slope, proximity to forest edge, proximity to large vegetated patch, proximity to town, trail density, on versus off linear features (roads, trails, and the railway), step length, turn angle, and movement state. We used backward stepwise selection with quasi-likelihood information criterion to select a top model for each species and season. We provide details about our covariates, R code, and parameter estimates in Additional files 2 and 3.

Animal resource selection and responses to anthropogenic development can also vary seasonally. Thus, we defined four seasons and created separate movement and SSF models for each species and season. We defined seasons based on animal movement, plant phenology, and human visitation rates. We classified *Spring* as May and June, which included plant emergence, ungulate parturition, grizzly bear mating, wolf denning, and moderate levels of human activity; *Summer* as July and August during the height of berry season and peak visitation; *Fall* as September and October when plants have senesced and the study area received moderate levels of visitation, and *Winter* as January and February for wolves with lower levels of visitation in backcountry areas and high levels of visitation near ski hills and towns.

#### Path simulation

We simulated individual-based carnivore movements from the hidden Markov movement models and SSFs across three landscape scenarios, from which we estimated changes in habitat patch, connectivity, and combined utilization distributions (UDs, Fig. 1). We thus adopted the Merriam approach for estimating connectivity as we combined movement behaviour with landscape structure to quantify individual-based estimates of movement success [7]. We simulated 144 million steps (200,000 paths, 60 days per path, and 12 fixes per day) within the 17,000 km^2^ study are for each species, season, and landscape scenario (Fig. 1, Additional file 1: Figure S1). Visual assessment of predicted habitat use remained stable from 100 to 144 million steps, which indicated that we simulated an adequate number of paths. We started paths at random locations that contained high quality habitat (the top third of SSF predictions). From each random location we generated 720 steps. Movement states and transition probabilities for each step depended on time of day, proximity to towns, and trail-road density. For each step, we calculated transition probabilities for each movement state, sampled *n* = 20 candidate states from the transition probabilities, and simulated 20 corresponding step lengths and turn angles from the state-specific movement parameters. We extracted environmental attributes of the 20 candidate locations and used the combination of environmental attributes, time of day, and their interactions with movement parameters to calculate probability of use for each location conditional on the 20 candidate locations. We probabilistically selected one of the candidate locations to use in the path and continued to the next step. We repeated this process for all steps in the path.

The study occurred in a rugged environment where steep, rocky mountain ranges can influence animal movements. We therefore defined unavailable habitat as barren and ice covered landscapes with slopes > 35 degrees, which were used by grizzly bears and wolves 3.9 and 0.5% of the time, respectively (Additional file 2: Table S4). We also classified towns and developed areas as unavailable habitat. To create realistic movement paths, we reduced the probability of simulated steps jumping across mountain ranges and towns by sampling four equidistant locations along proposed steps. We rejected steps if any of those locations occurred in the unavailable habitat. We minimized boundary effects on spatial predictions of use by terminating paths when > 40% of the proposed steps occurred outside the study area, by clipping out the outer 5 km of simulated steps, and by setting the study area boundary > 30 km from the towns of Banff and Canmore.

We simulated animal movements for three scenarios with varying levels of anthropogenic development: reference, current, and future. First, we removed the effect of towns, roads, trails, and the railway from SSFs when simulating paths under reference conditions, which we used as a null model of movement [25]. We removed the effects of anthropogenic development by setting β coefficients for those parameters to zero. Second, we simulated animal movements under current conditions from which we developed our SSFs. Finally, we simulated animal movements under one future scenario with expanded development and trail density. We modified the town of Canmore’s developed footprint to reflect residential and business development proposals in the 2020 Smith Creek and Three Sisters area structure plans [45, 46]. These proposals coupled with expanding recreational activities on surrounding trail networks have garnered extensive interest from the community about cumulative effects on carnivore movements and human-wildlife coexistence [40]. The proposals have been discussed for decades but have not been formally approved at this time. The developed footprint for the town of Banff is legally fixed under the National Parks Act and is not expected to increase. However, like many mountain towns, the creation and intensity of use on informal trails has increased near Banff and Canmore. We, thus added an inventory of informal trails to the existing formal trail network and recalculated metrics of trail density. Increased use of existing and new recreational trails has the potential to reduce wildlife connectivity [37, 47]. We simulated animal movements with the updated town and trail layers to estimate future connectivity. We included green spaces and golf courses as available habitat because carnivores can use these areas for movement.

#### Habitat use and connectivity

We created spatial predictions of habitat use (utilization distributions) for each species, season, and landscape scenario (Fig. 1). We counted the number of simulated locations that occurred within each 120 m × 120 m grid cell and then divided the counts by the number of total simulated locations [24]. The simulated paths contained interspersed slow and fast steps. We derived habitat use estimates for each movement state (slow and fast) and combined movement states to understand how anthropogenic development affected habitat quality associated with foraging-resting behaviour, travelling-hunting behaviour, and combined movements.

We assessed the performance of predicted habitat use by creating ten equal area bins from current habitat quality, tallying the proportion of GPS locations within each bin, and then calculating the Spearman rank correlation coefficient between the proportion of GPS locations and bin rank [48]. We calculated the Spearman rank correlation coefficient for each state (slow, fast, and combined) and for each individual animal as well as for all animals combined.

Next, we quantified the effects of anthropogenic development on the amount of high-quality habitat available to carnivores. We focused our analysis within a five km radius of the Trans Canada Highway between Banff and Canmore (366 km^2^). The five km radius represented the 0.99 and 0.95 quantiles of grizzly bear and wolf step lengths, respectively, and the focal study approximately covered the peak to peak width of the Bow Valley. We calculated the proportion of the study area classified as high-quality habitat (bin rank ≥ 7) for each movement species, season, time period, and movement state. We calculated percent intact habitat relative to reference conditions [47].

**Fig. 2:**
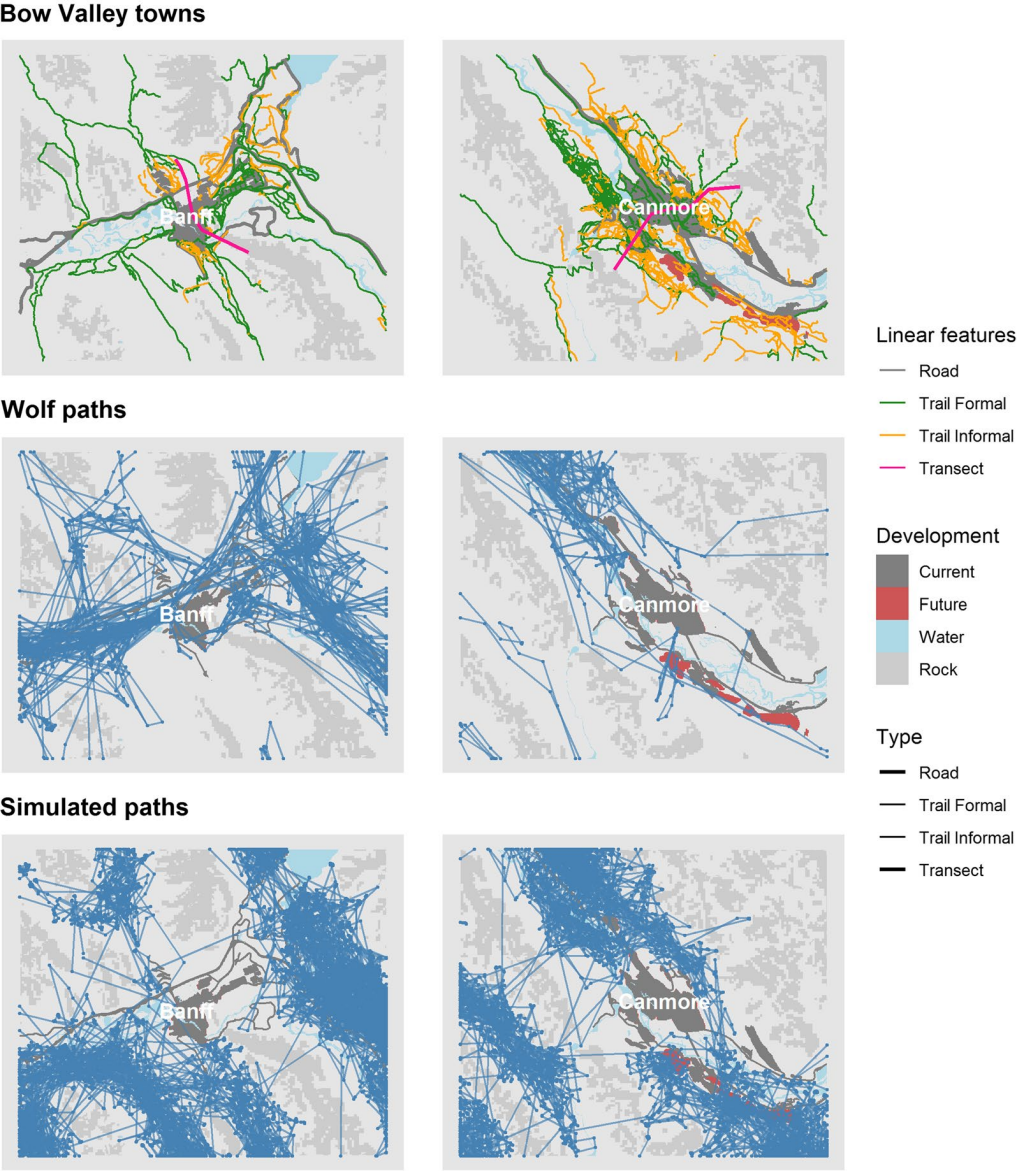
Illustration of anthropogenic development around the towns of Banff and Canmore, Alberta, observed wolf (*Canis lupus*) paths (winter), and a random sample of 800 simulated paths under current conditions. See Additional file 1: Figure S1 for a map of the entire study area and all GPS locations

Finally, we assessed two metrics of Merriam connectivity. First we counted the number of simulated paths that traversed 34 km between habitat patches west of Banff (Vermilion Lakes and surrounding area) and east of Canmore (Bow Valley Provincial Park and surrounding area). Second we counted the number of paths that crossed digital, cross-valley transects through the towns of Banff and Canmore (Fig. 2). We aligned transects so that they crossed the narrowest movement corridors under current condition, where the combination of rugged topography and development created pinch points to movement. We counted both the number of simulated paths that traversed habitat patches and crossed digital transects as our metric of connectivity because Merriam connectivity assesses movement success on a per animal basis [7]. We calculated connectivity as *n*_*traverse*_ / *n*_*reference*,_ where *n*_*traverse*_ was the number of unique paths that traversed patches and transects in current or future conditions and *n*_*reference*_ was the number of unique paths that traversed under reference conditions with no anthropogenic development. We evaluated how connectivity changed with species, season, and time period.

## Results

### Movement state

**Fig. 3:**
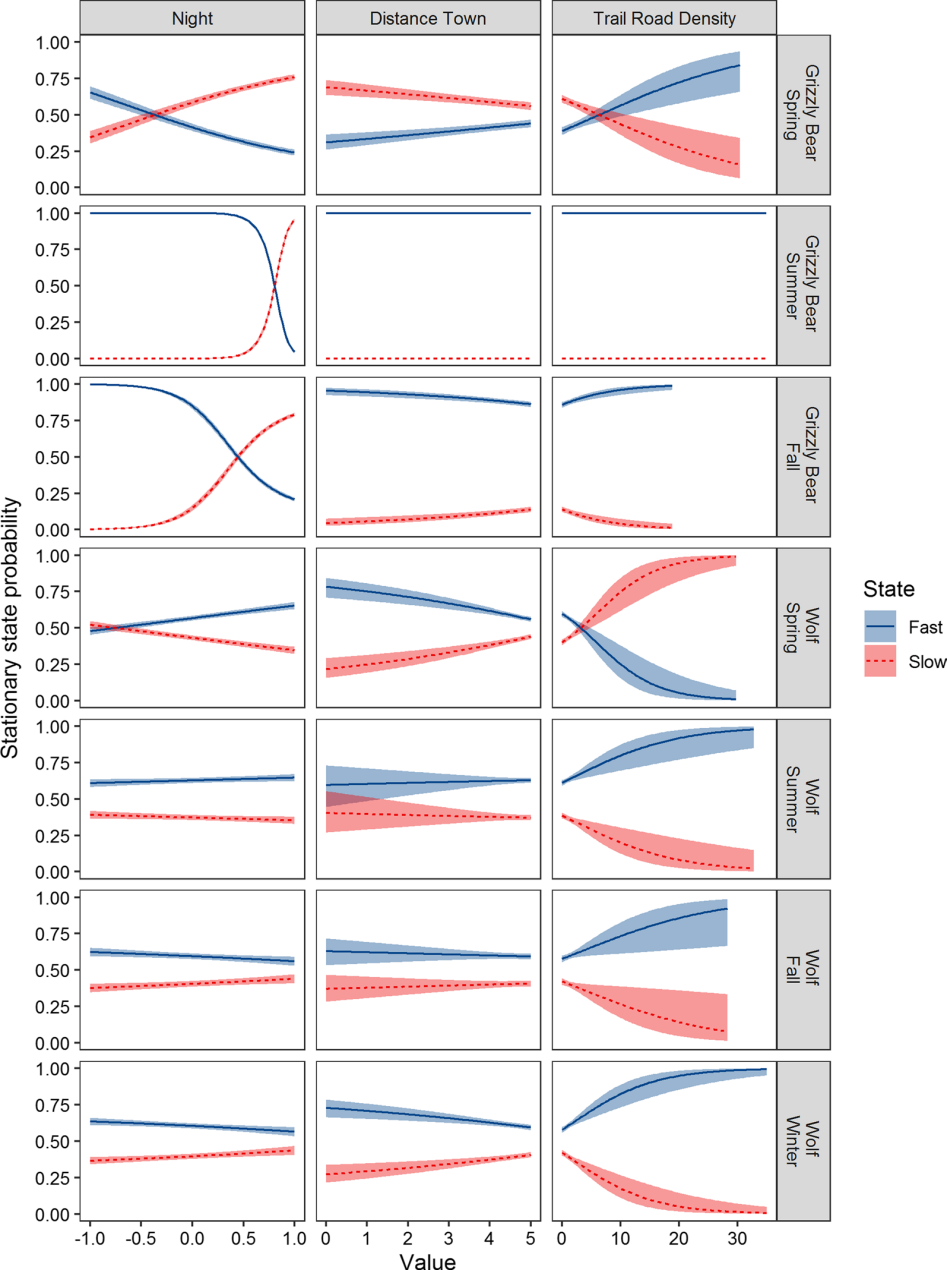
Grizzly bear (*Ursus arctos)* and wolf (*Canis lupus*) stationary state probabilities for slow and fast movements and 95% CI’s estimated from hidden Markov movement models. State depended on the previous state, distance to town (km), time of day (day = −1; night = 1), and trail-road density (km/km^2^). We interpreted slow and fast states to represent foraging-resting and travel behaviours, respectively

We analysed GPS data from 34 grizzly bears (19 females, 15 males, 72,217 locations) and 33 wolves (13 females, 20 males, 84,434 locations; Additional file 1: Figures S1–S3). Hidden Markov movement analysis found that grizzly bear and wolf movement states were indeed influenced as predicted by proximity to town, trail density, time of day, and their previous state (Fig. 3). The top model for both grizzly bears and wolves included a linear decay to five km and had lower AIC values compared to competing decay functions (ΔAIC ≥ 147.4 for grizzly bears and ΔAIC ≥ 3.5 for wolves). Grizzly bears and wolves were more likely to switch from a slow to a fast state within five km of towns in three out of the seven seasonal models with grizzly bears transitioning to faster states near towns in fall (e.g., parameter estimates for switching probabilities β_*SlowToFast*_ = -0.62 and β_*FastToSlow*_ = 0.91) and wolves in spring and winter (e.g., β_*SlowToFast*_ = -0.98 and β_*FastToSlow*_ = 0.08; Fig. 3; Additional file 2: Table S2). Grizzly bears and wolves were more likely to be in fast states of movement when in areas of high trail density in six of the seven seasonal models (e.g., grizzly bear summer β_*SlowToFast*_ = 0.25 and β_*FastToSlow*_ = -0.04; wolf summer β_*SlowToFast*_ = -0.02 and β_*FastToSlow*_ = -0.24; Fig. 3). The exception occurred in the spring when wolves were more likely to switch to slow movement rates in areas of high trail density, possibly because their prey species like elk and deer congregated in valley bottoms to access early emergent vegetation and because some den and rendezvous sites were located within 500 m of trails and roads. Grizzly bears had a much stronger diurnal cycle of movement states than wolves (Fig. 3). Grizzly bears increased their proportion of time in slow states at night. Wolves had a weaker and sometimes opposite diurnal cycle. Wolves increased the proportion of time in slow states at night during fall and winter only. Wolves increased the proportion of time in fast states at night during spring and summer, which coincided with the longest days of the year. Grizzly bears predominately travelled in their fast state in summer except near midnight.

### Step selection

**Fig. 4:**
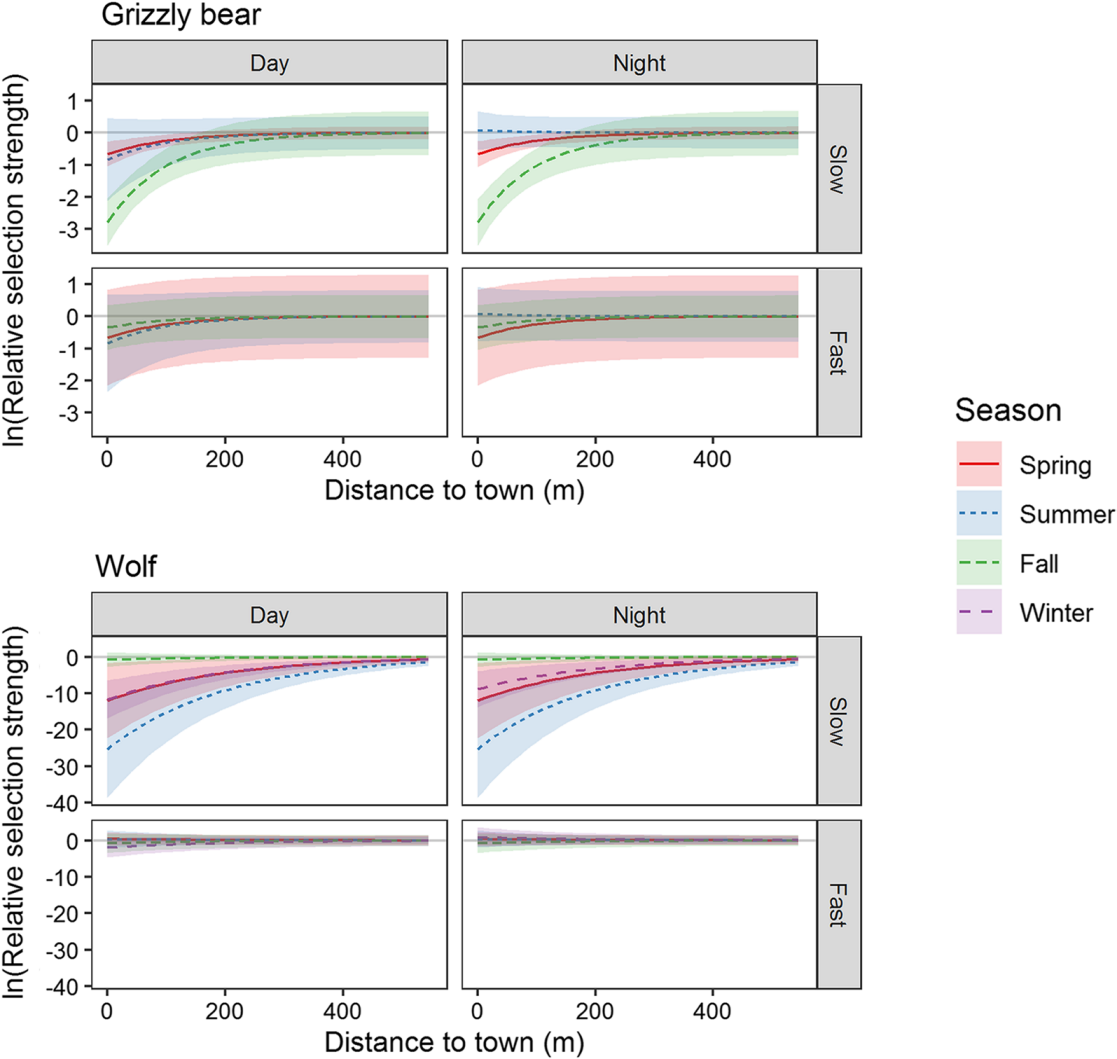
Grizzly bear (*Ursus arctos)* and wolf (*Canis lupus*) relative selection strength (RSS) and 95% CI’s for proximity to town. Carnivores avoided areas near towns especially in their slow state during the day. Avoidance waned for fast states of movement

**Fig. 5:**
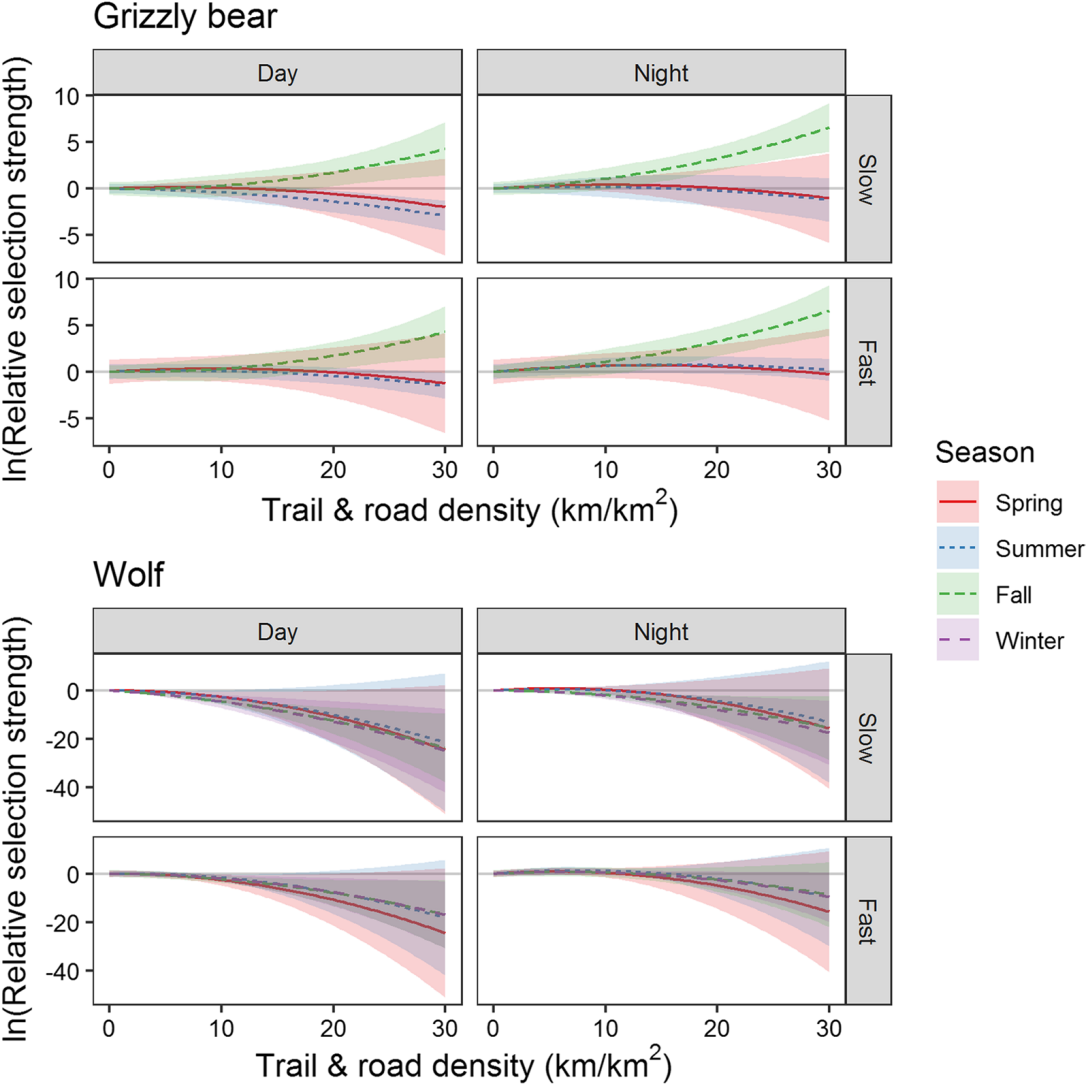
Grizzly bear (*Ursus arctos)* and wolf (*Canis lupus*) relative selection strength (RSS) and 95% CI’s for trail and road density. Carnivores avoided areas of high trail density especially in their slow state during the day. Avoidance waned for fast states and at night. Grizzly bears selected high trail density during the fall

**Fig. 6:**
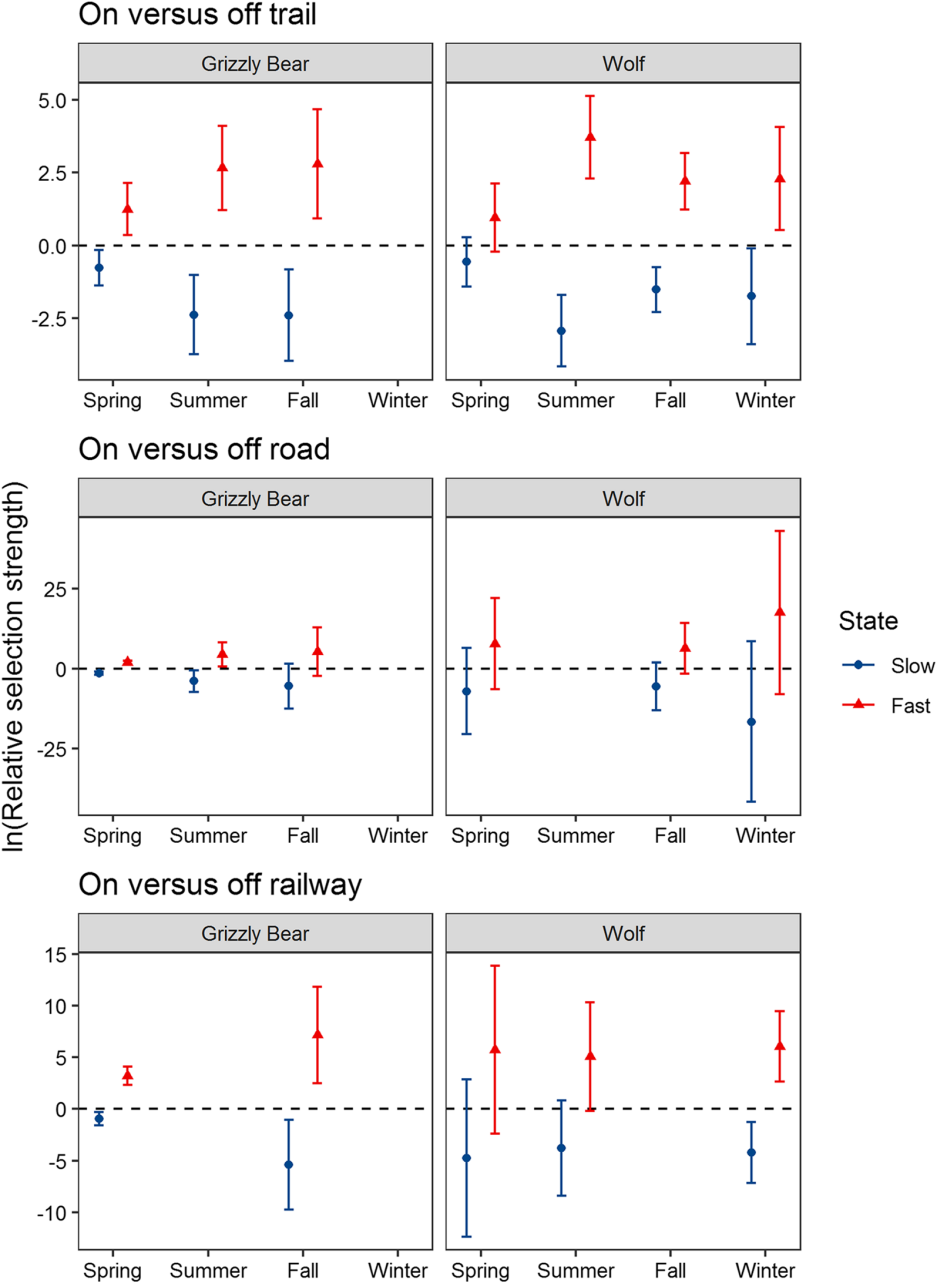
Grizzly bear (*Ursus arctos)* and wolf (*Canis lupus*) step selection function β coefficients (ln[relative selection strength]) and 95% CI’s for carnivore selection of trails, roads, and the railway. The model selection process excluded linear features in some seasons. Carnivore selection for linear features increased from slow to fast states of travel

The SSF analysis indicated that wolves and grizzly bears generally avoided anthropogenic features, especially when foraging and resting in their slow state of travel (Figs. 4, 5, and 6, Additional file 2: Table S3). Wolves and grizzly bears avoided areas near town in their slow state during the day with wolves exhibiting stronger avoidance than grizzly bears. Wolves avoided areas within 400 to 500 m of towns (median β = −8.99, range from −26.8 to −0.55) and grizzly bears avoided areas within 200 to 300 m of towns (median β = −0.77, range from −3.26 to −0.23). Both species had a higher tolerance for areas near town and areas with high trail-road density when travelling in their fast state and at night, with movement state having a larger effect than time of day. Overall, towns had minimal effects on animals for fast states of travel.

We found a quadratic effect of trail and road density on wolf and grizzly bear step selection in all models except for grizzly bears in fall (Fig. 5). Wolves showed stronger avoidance of high trail and road density (β_quadratic_ range from −0.13 to −0.04) compared to grizzly bears (β_quadratic_ = −0.02 for both spring and summer). Wolves avoided high trail and road density during all seasons and times of day with the strongest avoidance occurring in slow states during the day. Grizzly bears selected moderate trail and road densities and avoided high densities during the spring and summer but not the fall when they selected areas with high trail and road density.

Grizzly bears and wolves avoided trails, roads, and the railway while in their slow state and selected these features as travel routes in their fast state (Fig. 6). The state dependent selection for these three features occurred in all but two seasonal models. Grizzly bears and wolf selection for linear features in fast versus slow states of movement were positive for trails (e.g., coefficients for fast state versus slow state β_*GrizzlySpring*_ = 1.25, SE = 0.21; β_*WolfSpring*_ = 0.96, SE = 0.19), the railway (e.g., β_*GrizzlySpring*_ = 3.22, SE = 0.25; β_*WolfSpring*_ = 5.73, SE = 2.28), and roads (e.g., β_*GrizzlySpring*_ = 1.91, SE = 0.22; β_*WolfSpring*_ = 7.77, SE = 2.06). Grizzly bears showed strong selection for the railway in spring and fall but not summer (Fig. 6). Wolves showed the clearest selection for railways in winter when deep snow was more likely to impede travel. While grizzly bears and wolves selected linear features as travel routes, they also travelled extensively through the broader landscape as only 6 and 4% of their travel steps respectively occurred within 30 m of linear features.

### Path simulation

Simulated paths under reference, current, and future land use scenarios had similar movement attributes compared observed paths (e.g., Fig. 2). Both simulated and observed paths contained series of short steps with high turn angles interspersed with long distance movements and strong directional persistence. Under reference conditions, simulated paths were concentrated in the valley bottoms and used areas within the current footprint of towns. The combination of towns and rugged topography cumulatively constrained the movements of both observed and simulated paths under current and future scenarios.

We assessed model fit of habitat use predictions derived from simulated paths. We calculated the Spearman rank correlation coefficient between number of GPS locations within each habitat use bin under current conditions and bin rank. We assessed model fit for habitat use predicted from slow, fast, and combined states of movement. Overall, models had high correlation between bin rank and number of GPS locations (Additional file 2: Figure S1). Spearman correlation coefficients for the pooled data sets were on average higher for wolves (mean = 0.99) than grizzly bears (mean = 0.90). Wolf models performed well for all individuals, whereas performance of the grizzly bear models varied considerably among individuals, especially during summer and fall. Model performance for fast and slow states were similar, except for grizzly bears in summer where the fast state predictions had better fit than the slow state predictions.

### Habitat use and connectivity

**Fig. 7:**
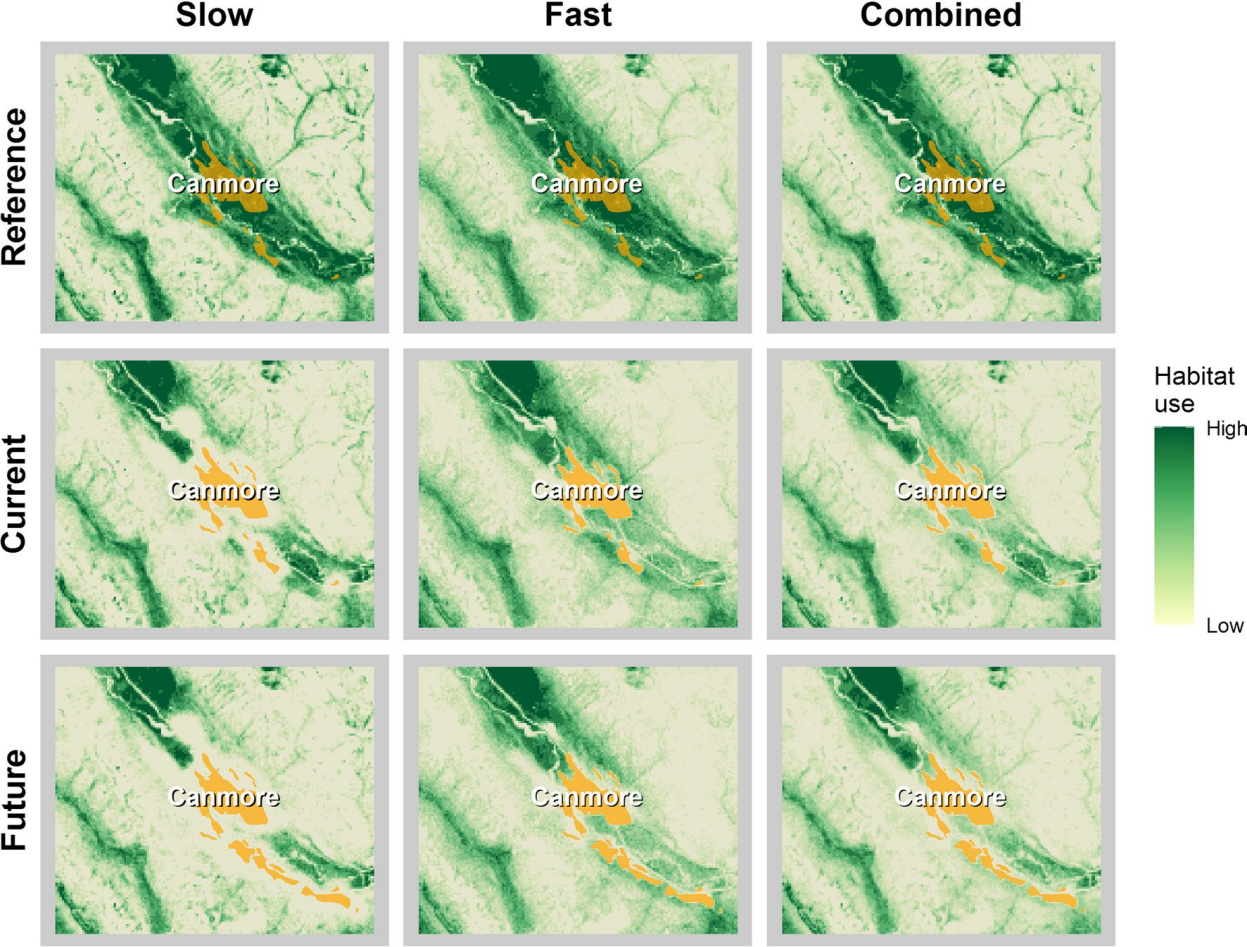
Maps showing wolf (*Canis lupus*) predicted habitat for slow, fast, and combined states of movement around Canmore, Alberta during summer. Habitat use was derived from simulated paths. Habitat quality decreased around Canmore from reference to current and from current to future conditions, especially for slow states of movement associated with foraging and resting

Habitat use predictions under current and future conditions showed a cumulative decrease in use around the towns and in areas of high trail and road density relative to reference conditions (except grizzly bears in fall). Slow states had higher levels of habitat degradation quality compared to fast states (Fig. 7). This resulted in minimal slow state habitat (e.g., resting, foraging) around the towns of Banff and Canmore (Fig. 7, Additional file 1: Figures S4 to S7). Corridors of fast state use (e.g. travelling) remained around some sections of towns. Habitat degradation around towns was more severe for wolves than grizzly bears.

**Fig. 8:**
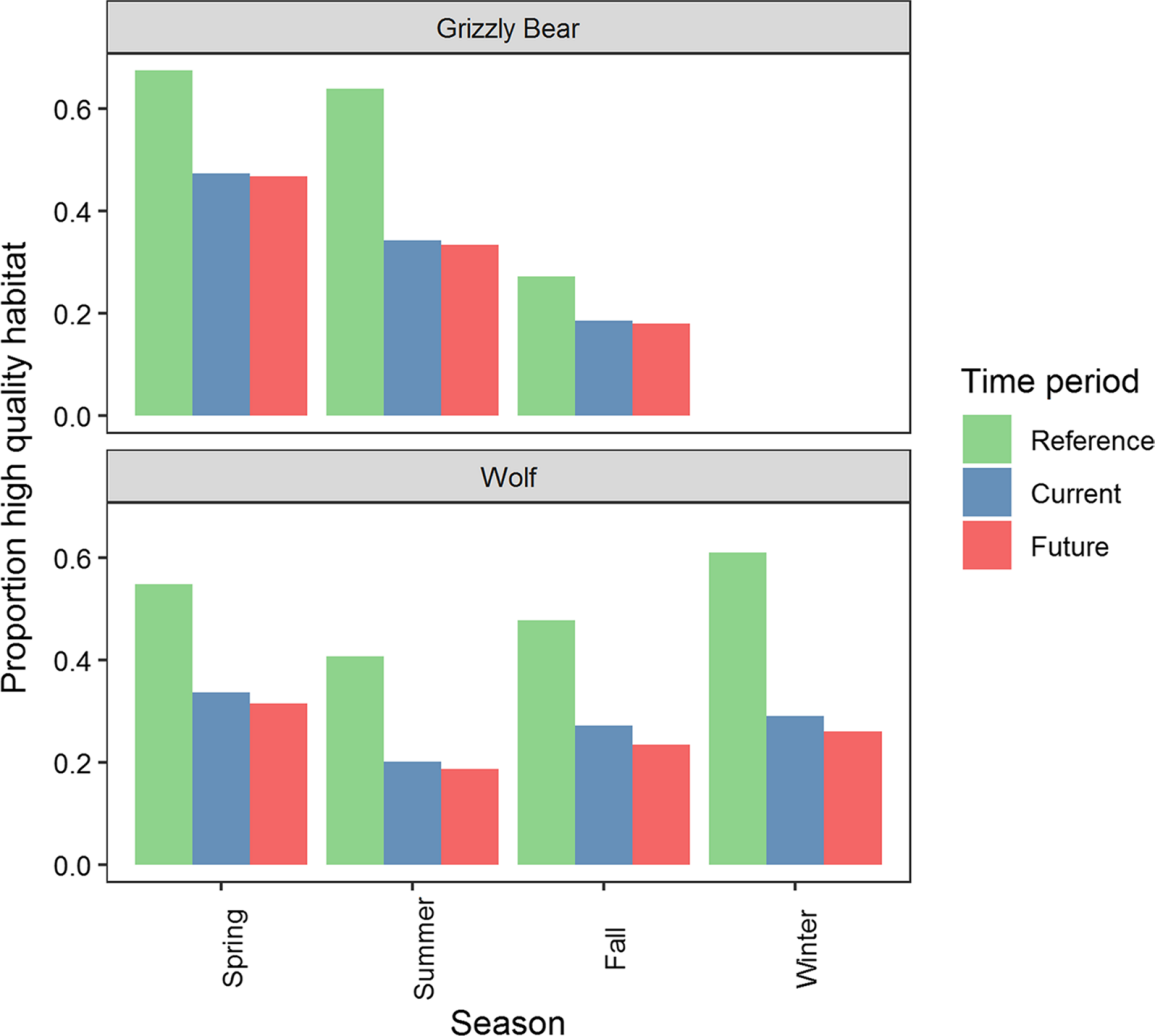
Proportion of the Bow Valley between Banff and Canmore, Alberta classified as high-quality habitat (bin rank  ≥ 7) for combined fast and slow movement states under reference, current, and future land use scenarios. We simulated 144 million paths for each species, season, and time period and used the density of locations and equal area binning to predict habitat use

The proportion of the Bow Valley classified as high-quality habitat for combined states of movement decreased from reference to current conditions by an average of 0.20 and 0.24 for grizzly bears and wolves respectively (Fig. 8). This equated to 36% and 46% losses of available high-quality habitat. The proportion of the valley classified as high-quality habitat further decreased from current to future conditions by 0.01 and 0.03 for grizzly bears and wolves respectively, which resulted in a total loss of 37% and 51% of high-quality habitat relative to reference conditions. Habitat degradation was highest in summer and lowest in the spring and fall for grizzly bears. Habitat degradation was highest for wolves in winter when deep snows confine movements to valley bottoms. Changes in high-quality habitat accounted for both decreased use near anthropogenic developments and concurrent increased use in the surrounding area as simulated animals spent more time in less developed portions of the landscape.

**Fig. 9:**
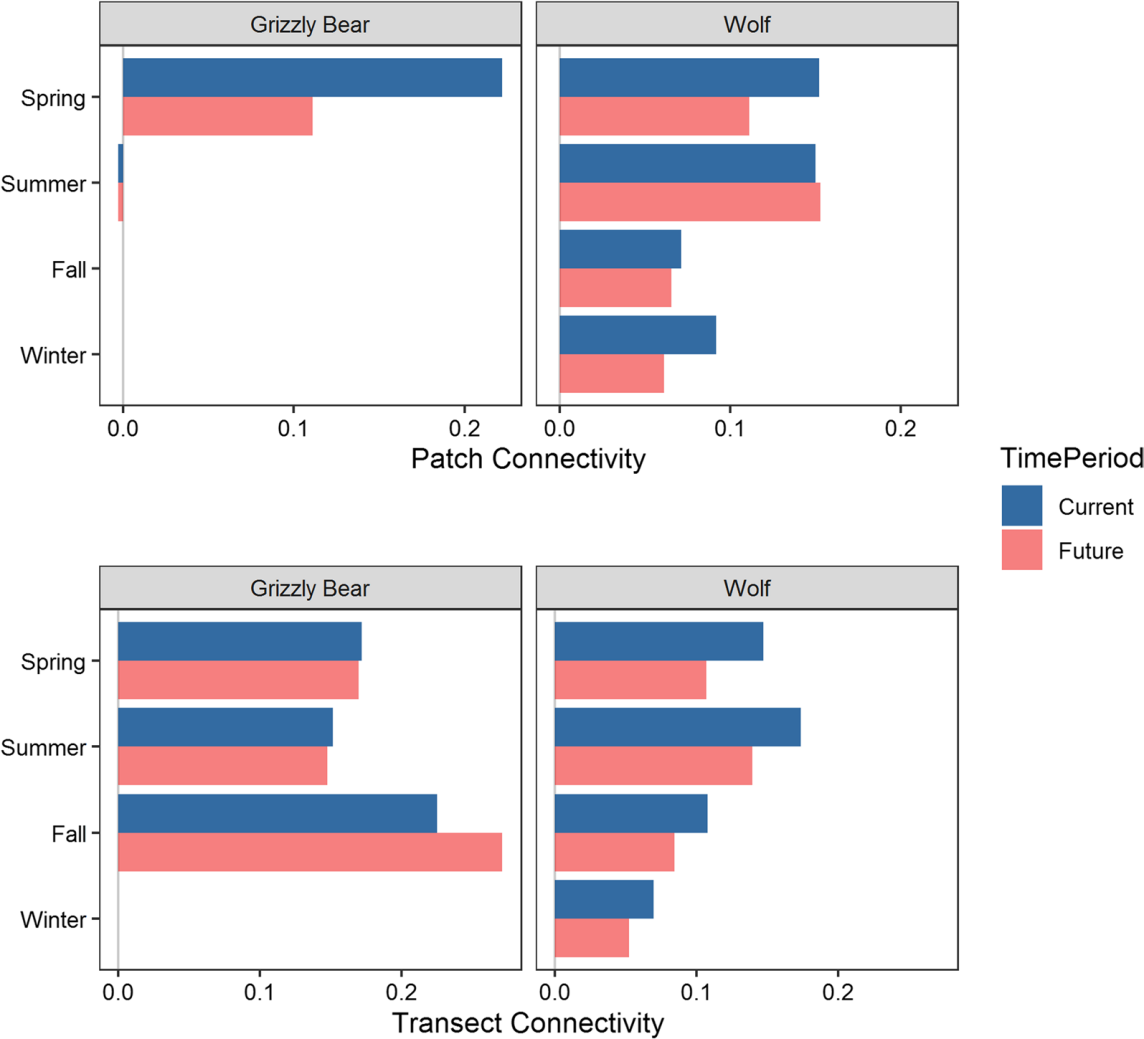
Merriam connectivity estimates for grizzly bears and wolves between habitat patches west and east of Banff and Canmore respectively (Patch Connectivity) and across valley wide transects through the towns of Banff and Canmore (Transect Connectivity). We estimated connectivity for current and future footprints of anthropogenic development by comparing the number of simulated paths that traversed patches and transects under current and future conditions to crossing rates from reference conditions. There were no winter estimates for grizzly bears because they hibernated at that time.

Merriam connectivity between habitat patches west of Banff and east of Canmore under current conditions averaged 11% for both grizzly bears and wolves (Fig. 9). Patch connectivity declined to 5% for grizzly bears and 10% for wolves under future conditions. Patch connectivity estimates were more reliable for wolves because wolves had faster movement rates and > 300 reference paths traversed the 34 km between the two patches for each simulation (Additional file 2: Table S5). In contrast, less that ten grizzly bear reference paths travelled between the two patches due to their slower movement rates. Merriam connectivity measured on valley wide transects near Banff and Canmore had thousands of reference path crossings. Transect connectivity ranged between 6 and 25% under current conditions with mean values of 18% for grizzly bears and 12% for wolves (Fig. 9). Grizzly bear connectivity increased an average of 1% from current to future, with the average increase being driven by fall movements. Wolf connectivity further declined an average of 3% from current to future conditions. Grizzly bear connectivity was highest in the fall, when they selected for areas with high trail and road density. Wolf connectivity was highest in summer. Future developments had stronger negative effects on wolf connectivity than on grizzly bear connectivity. Overall, patch and transect connectivity declined an average of 85% (SD = 5%) compared to reference conditions.

## Discussion

Our study was novel in that we combined hidden Markov models with SSFs to assess the influence of current and future anthropogenic development on multiple movement processes including state-dependent movement behaviour, habitat use, and connectivity. We found that the cumulative effects of anthropogenic development caused more extensive habitat degradation for slow movements than for fast movements. Overall, grizzly bears and especially wolves increased their speed of travel near anthropogenic development to minimize encounters with people and avoided anthropogenic development for slow movements, especially during the day when people were more active. This habitat degradation reduced the amount of high quality habitat available for foraging and resting, and thus reduced the ability of carnivores to regulate prey populations that seek human settlements as prey refuges [33] and reduced the functionality of wildlife corridors [5]. We found that that towns, roads, and trails together reduced connectivity from reference conditions around mountain towns by an average of 85%. Our study supports the growing body of research showing the negative effects of anthropogenic development on wildlife movements [e.g., 49–51.

Anthropogenic development in our study increased transitions rates from slow to fast movements for both grizzly bears and wolves in all seasons except for grizzly bears in summer when they spent most of their time in fast states of movement and for wolves in spring when some packs denned near trails and roads. Globally, human activity has variable effects on animal movement, including movements of large carnivores [50]. In many cases, human activity has reduced movement rates of animals through barrier effects or by providing resource rich environments for concentrated foraging [49]. For example, puma in California exhibited slower movement rates near anthropogenic developments, perhaps because they were forced to travel in rugged terrain that slowed movements [52]. Similarly, wild dogs in Africa decreased movement rates near human settlements but increased rates of travel outside of protected areas, perhaps because of lower prey availability [19]. Conversely, African lions (*Panthera leo*) increased their speed of travel near bomas (livestock enclosures), perhaps to reduce their risk of encountering and being detected by people [52]. The combined movement models and step selection functions from our study suggest that grizzly bears and wolves sped up movement rates near anthropogenic developments due to a combination of factors including increased encounter rates with people and reduction in secure habitat for foraging and resting near towns and areas with high densities of trails and roads. These models also suggest that grizzly bears and wolves use linear features to increase travel efficiency, but this can often subject them to increased mortality risk due to vehicle collisions, human hunting and management actions [34, 53].

Grizzly bear and wolf resource selection responses to anthropogenic development depended both on behavioural state and time of day, with larger effect sizes for behavioural state. Our results were consistent with the few studies to assess the effects of movement state and time of day on resource selection [15, 54]. Like grizzly bears and wolves in our study, African lions avoided human activity during the day when foraging and resting, yet had higher tolerance for human activity when travelling and at night [15]. Our results are consistent with other research showing that wildlife are more likely to use habitat and travel through areas with people at night than during the day [54, 55]. For example, in a meta-analysis Gaynor et al. [55] found that many taxa adapted to human disturbance by increasing their activity at night by an average factor of 1.36. This nocturnal temporal shift in movement and foraging behaviour allows animals to access habitat required to maintain fitness while minimizing encounters with people.

Grizzly bears and wolves in our study avoided areas near towns when in slow movement states even though towns contained attractive natural and anthropogenic food sources [34, 56]. For instance, elk, which are an important prey species for grizzly bears and wolves, congregated and calved near towns to reduce predation risk [33]. Even with this attractive food source, grizzly bears avoided areas 200 to 300 m from towns while wolves avoided areas 400 to 500 m from towns. Avoidance of towns tapered at night and was negligible for wolves in their fast state of movement. Together, this suggests towns had stronger effects on habitat required for foraging and resting compared to connectivity habitat required for travel.

Grizzly bears and wolves both avoided areas with high trail and road density when in slow states of movement, likely to reduce encounter rates with people. The exception occurred in the fall, when grizzly bears selected areas with high trail densities. Trails at this time of year had low levels of visitation and grizzly bears likely selected for seasonal foods associated with high trail density. For example, *Sheperdia canadensis* berries and *Hedysarum* spp. roots are important food sources for grizzly bears in the summer and early fall and can be found along forest edges and in open canopy forests that receive higher levels of solar radiation [57]. While grizzly bears selected areas with higher trail density in the fall, their selection for these features likely depends more on the combination of available foods and levels of human activity than on the trails themselves.

Our study could be improved with better estimates of recreational activity on trail networks [40]. While we used trail and road density as a surrogate for intensity of human use, carnivores typically avoid encounters with people rather than the physical density of linear features [38]. Recent studies show promising approaches for predicting recreational activity by directly tracking recreationists’ movements [47], inferring activity from mobile device and crowdsourced data [58, 59], or modelling spatial and temporal trends in trail use [60]. Stronger links between recreational activity and wildlife movements would improve our understanding of recreational thresholds for wildlife and our ability to manage human-wildlife coexistence [61, 62].

Numerous studies have found that grizzly bears [63, 64] and wolves [36, 62, 65] avoid human activity, which can contribute to the fragmentation of populations [66, 67]. However, few studies have compared the behaviour of the two species. Wolves in our study exhibited stronger avoidance of towns and areas of high trail-road density relative to grizzly bears. The muted response of grizzly bears was likely influenced by high individual variability in responses to anthropogenic development [60, 68]. Model fit from the predicted habitat use had high variability among individual grizzly bears (but not wolves), which reflects high individual variability in resource selection. Thus, our results for grizzly bears likely averaged results from both wary and habituated individuals. This could lead to an underestimate of the effect of human activity on surviving bears because habituated bears have dismal survival prospects in busy landscapes such as the Bow Valley [34]. Simulating movements from random coefficients could highlight estimates of connectivity for both wary and habituated animals and could help identify areas likely to have high levels of human wildlife conflict [34, 69]. Finally, grizzly bears are highly motivated to find food, including human and natural foods in and around residential areas, which can lead to increased human-wildlife conflict [56]. As such, grizzly bears that use areas near people face high risk of mortality, which can lead to population level source-sink dynamics [34, 70]. Pairing demographic outcomes such as survival and reproduction with individual-level behavioural responses to human activity could help bridge the gap to what Fahrig et al. [7] identified as one of the missing links in connectivity science, population-level connectivity.

Integrated step selection analyses that interact movement parameters with anthropogenic features could also be used to estimate the effects of human activity on habitat use and connectivity without the added step of developing hidden Markov movement models [24, 29]. However, we found that classifying movements into discrete behavioural states simplified our interpretation about how human activity affected movement processes. Moreover, animal motivations to move include accessing habitat required for fitness enhancing behaviours such as foraging, resting, and reproduction [71]. Understanding and conserving habitat for slow-state behaviours could affect realized movement rates and could have important consequences for fitness and population level-connectivity.

## Conclusions

Our results highlight the adverse effects of anthropogenic development on habitat use and connectivity with more pronounced effects on habitat required for foraging and resting. Restoration actions, such as removal of human footprint, managing or consolidating recreational activity, and trail closures have potential to improve habitat quality and population-level connectivity. Wildlife have responded to restoration actions by increasing their use of corridors and degraded habitat following reductions in human activity, both in our ecosystem [38, 72, 73] and around the world [74]. For example, early work in our study area demonstrated positive wildlife connectivity consequences of removing recreational footprint in the Cascade wildlife corridor on the north side of the Banff town site [72], and positive effects of a temporal road closure on wildlife habitat quality [38]. Our approach for simulating animal movements could be applied to assess the effects of potential restoration actions on behavioural-specific habitat use and connectivity [6, 75, 76]. Simulations and restoration actions could focus on highway mitigations [77], reductions in trail density, permanent closures, seasonal closures, or temporal closures [38]. In the face of global increases in human activity, especially surrounding parks and protected areas [39], proactive habitat protection and restoration actions will be required to maintain habitat quality and connectivity for wide ranging wildlife [1].

## Supplementary Information

**Additional file 1**. Maps of study area, observed GPS locations, and predicted habitat use from state-dependent step selection function models.

**Additional file 2**. Parameter estimates from hidden Markov movement models and step selection functions.

**Additional file 3**. Wolf summer data and R scripts.

## Data Availability

All data generated or analysed during this study are included in this published article and in its supplementary information files. We provide wolf summer data and R scripts used to fit hidden Markov movement models, fit step selection functions, and simulate paths in Additional file 3.
