# Supplementary material for: Towns and trails drive carnivore movement behaviour, resource selection, and connectivity
Source: Mov Ecol. 2022 Apr 8;10:17. doi: 10.1186/s40462-022-00318-5 (PMC8994267; doi:10.1186/s40462-022-00318-5)
Supplement: Supplementary file 1 — Additional file 1. Maps of study area, observed GPS locations, and predicted habitat use from state-dependent step selection function models. [file 40462_2022_318_MOESM1_ESM.docx]

**Towns and Trails Drive Carnivore Movement Behaviour, Resource Selection, and Connectivity**

J. Whittington, M. Hebblewhite, R. Baron, A.T. Ford, J. Paczkowski

Movement Ecology

# Appendix S1. Maps of study area, observed GPS locations, and predicted habitat use from step selection function models.

## Section S1.1 Study area and GPS locations

Research Permits: Researchers fit wolves and grizzly bears with GPS collars for several research projects with the following permits: University of Alberta Animal care protocol ID# 353112, University of Montana Institutional Animal Care Protocol 059-08MHECS-120908, 004-16MHECS-020916, 066-18MHWB-123118, Parks Canada Research and Collection Permits LL-2010-4392, BAN-2015-18276, BAN-2018-30898, LL- 2012-10975.


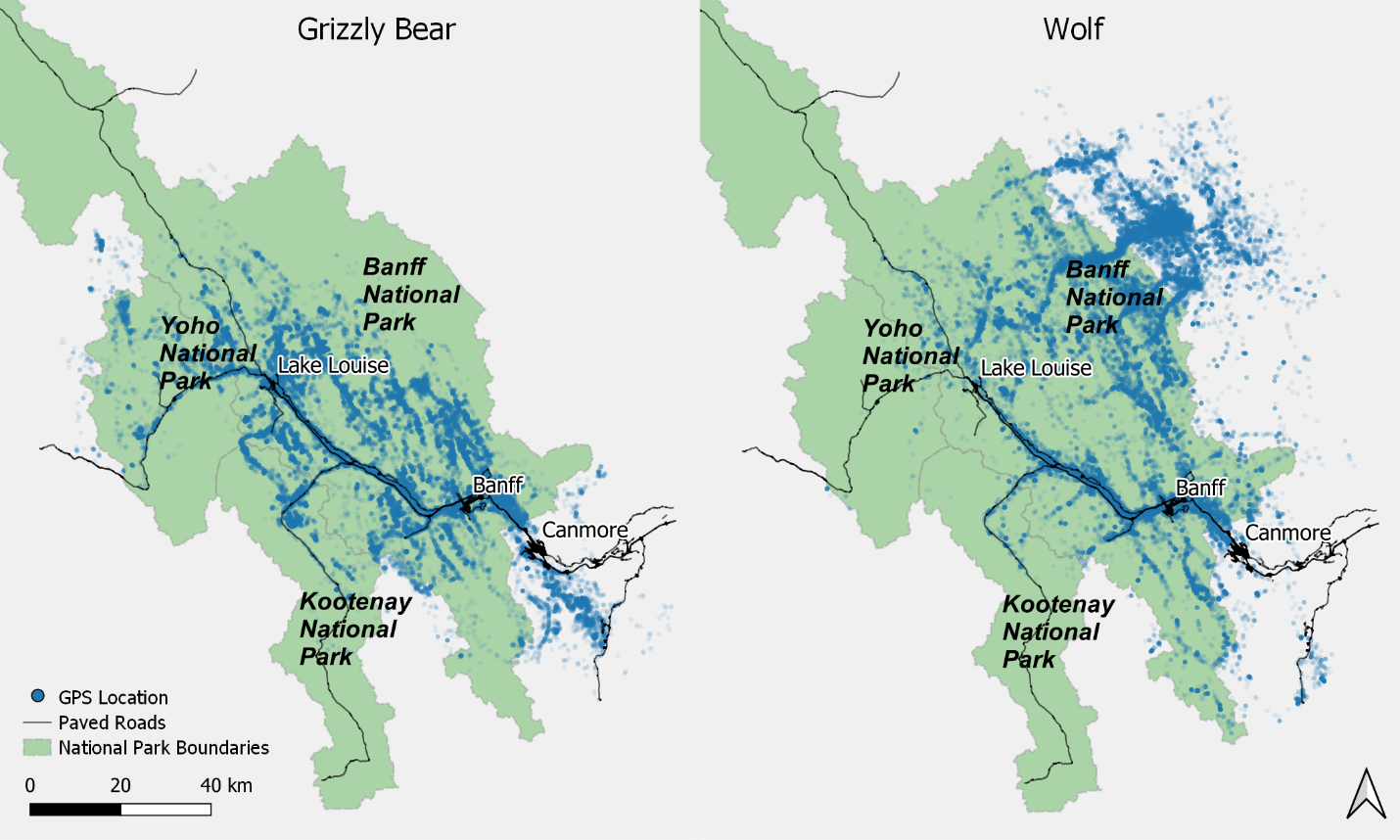


Figure S1. Grizzly bear and wolf GPS locations within and adjacent to Banff, Kootenay, and Yoho National Parks of Canada, 2000 to 2020. We used GPS locations from this study area to develop hidden Markov movement models and step selection functions.


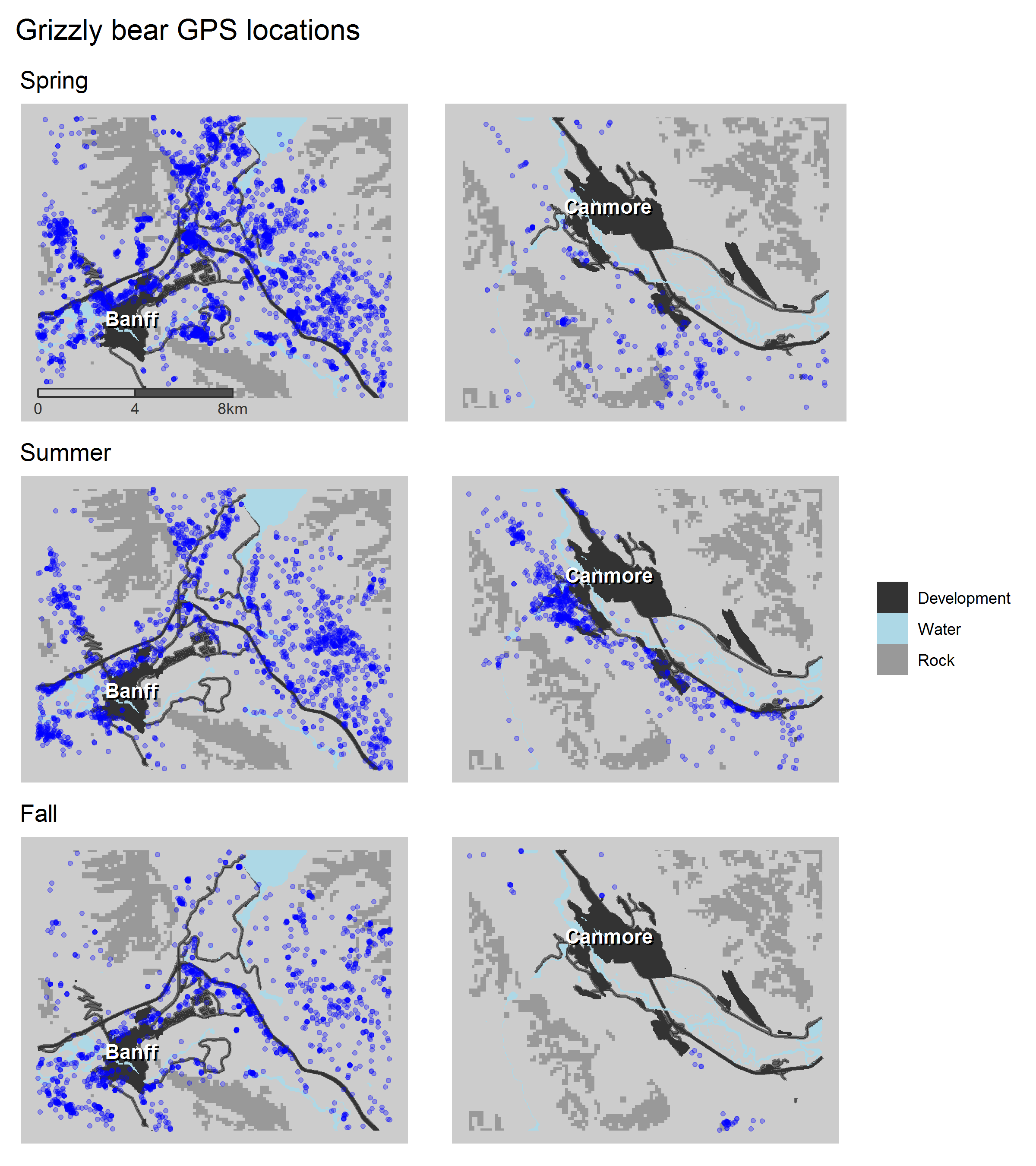


Figure S2. Distribution of grizzly bear locations around the towns of Banff and Canmore by season.


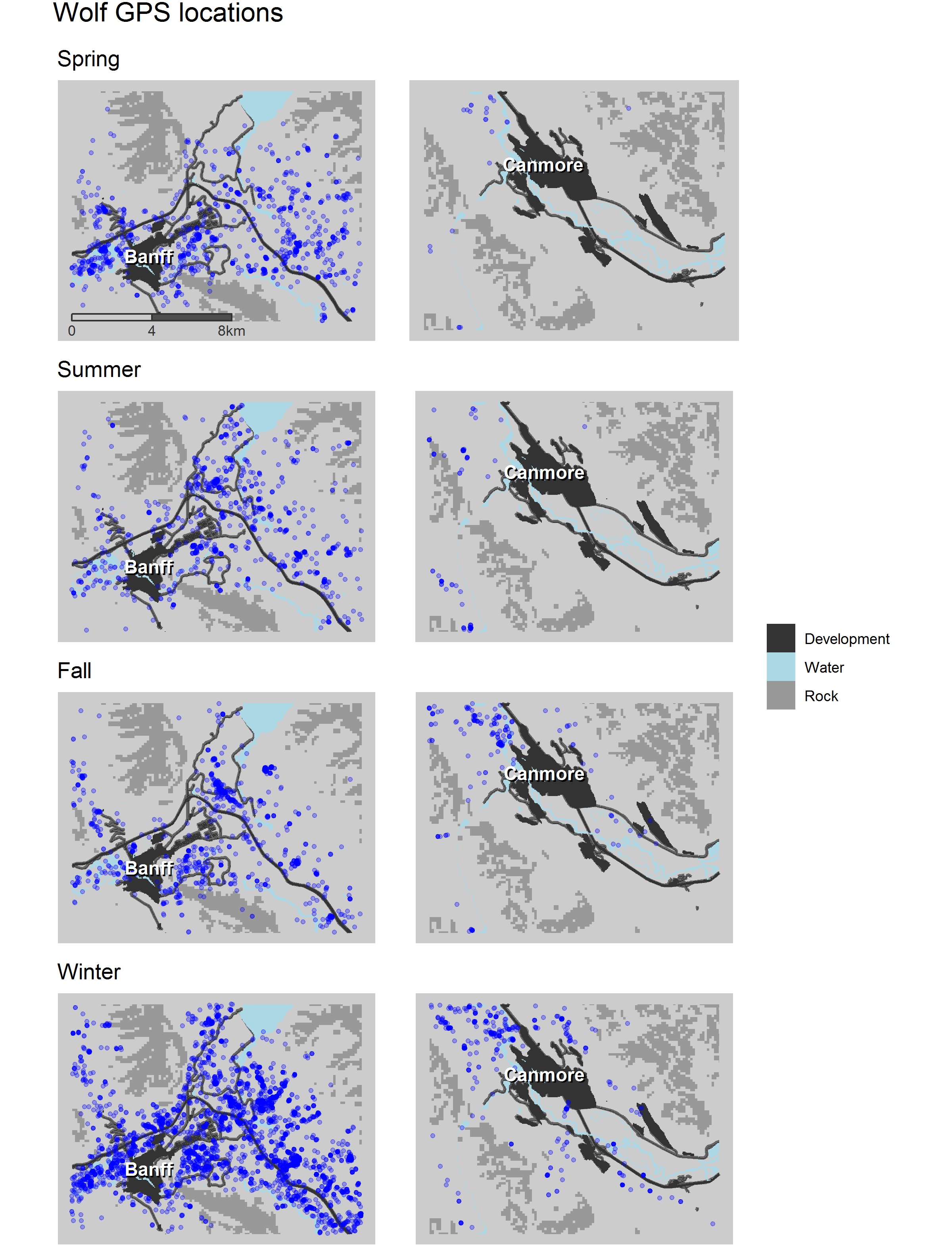


Figure S3. Distribution of wolf locations around the towns of Banff and Canmore by season.

## Section S1.2 Grizzly bear and wolf summer predicted habitat use by movement state and landscape scenario for Banff and Canmore, Alberta, Canada.


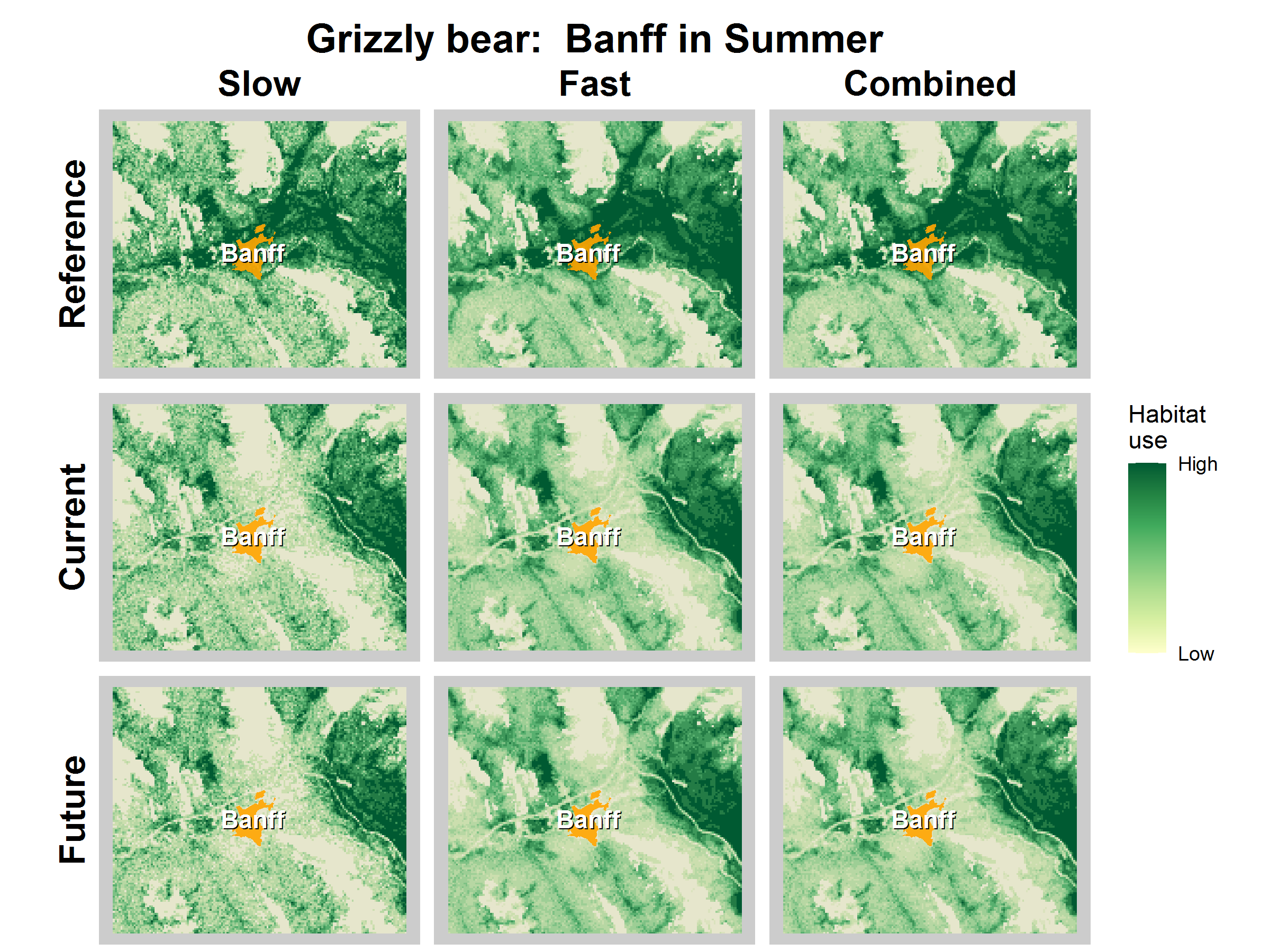


Figure S4. Grizzly bear predicted habitat use by movement state and time period around Banff, Alberta.


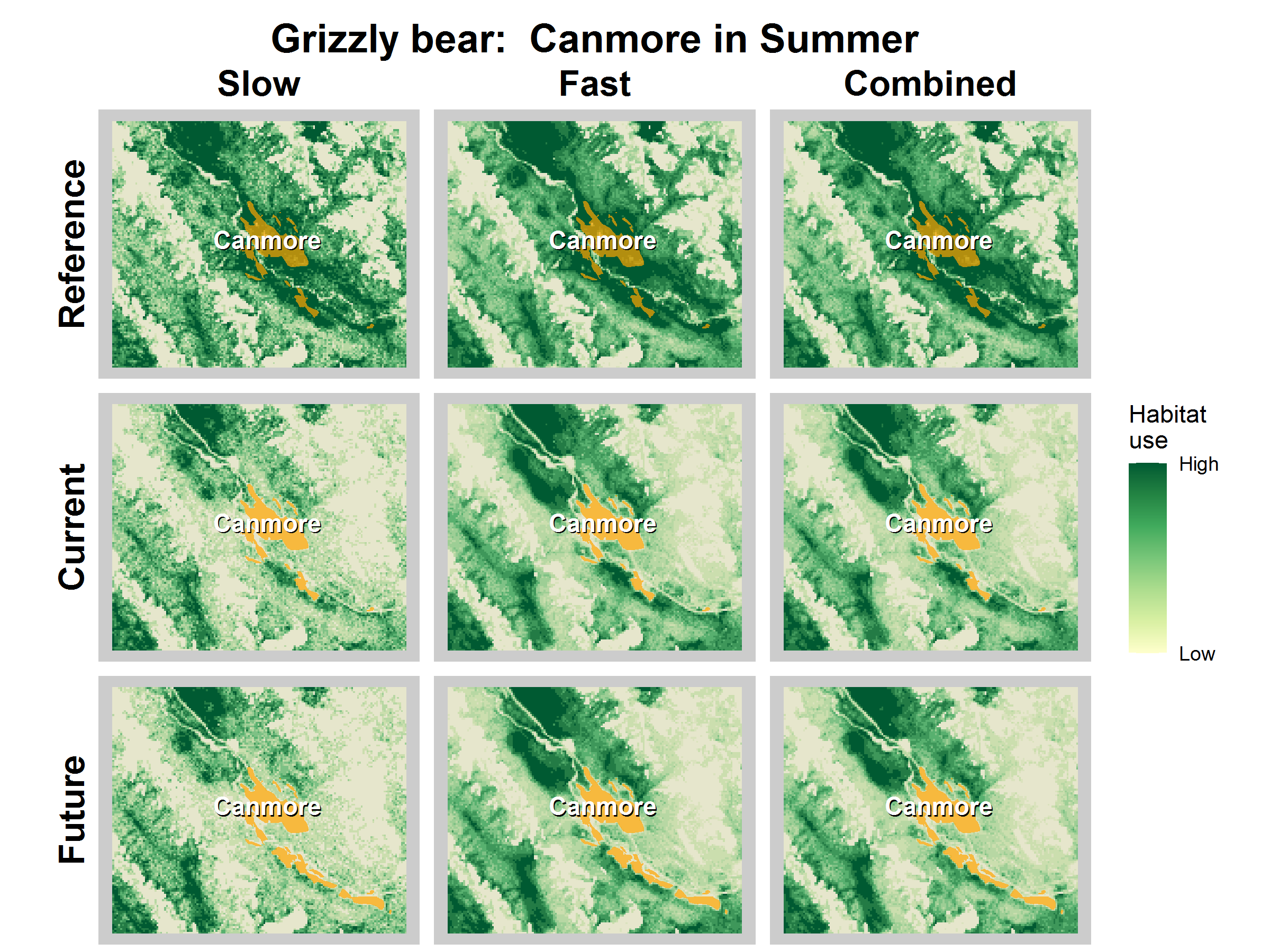


Figure S5. Grizzly bear predicted habitat use by movement state and time period around Canmore, Alberta.


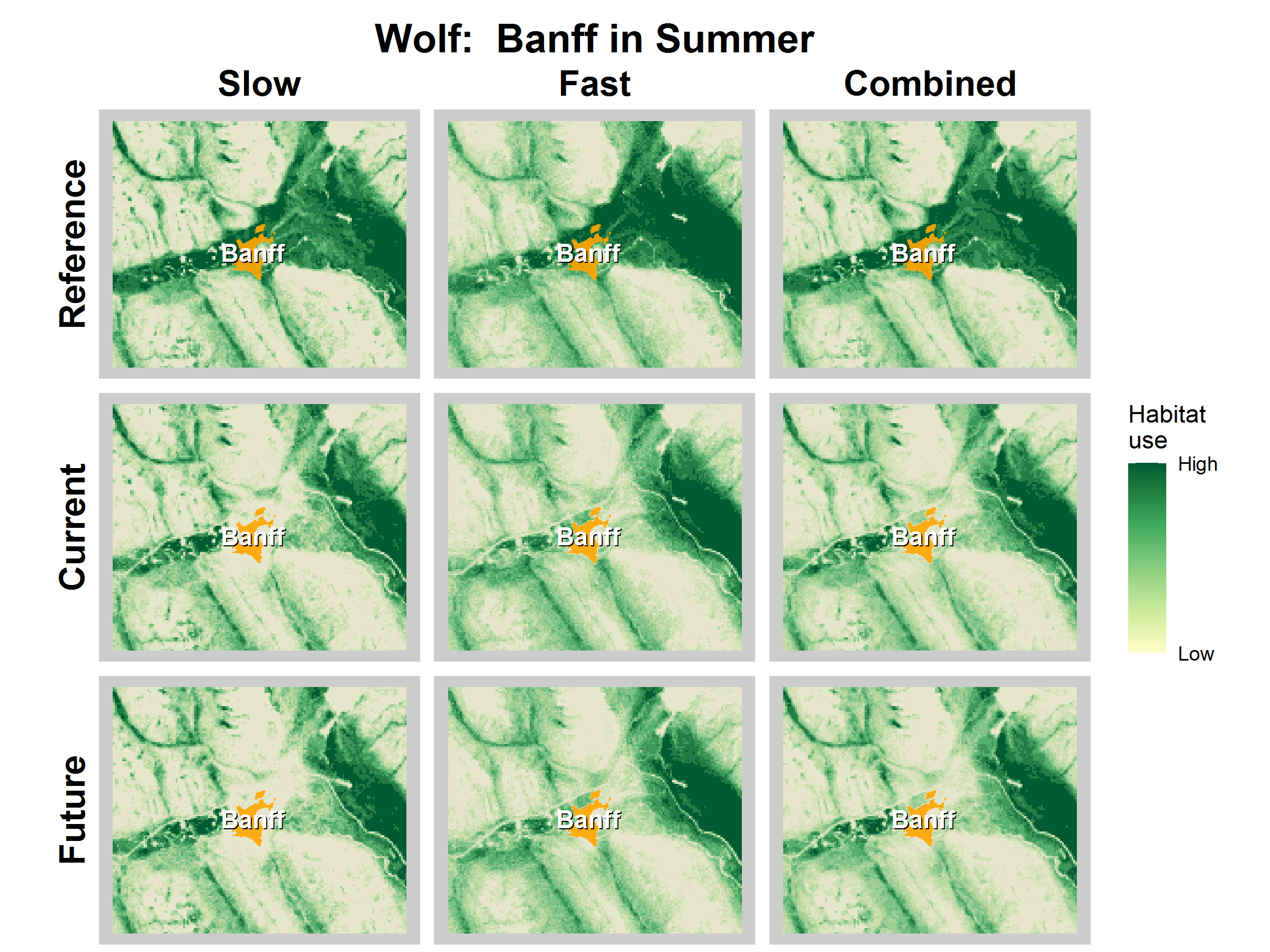


Figure S6. Wolf predicted habitat use by movement state and time period around Banff, Alberta.


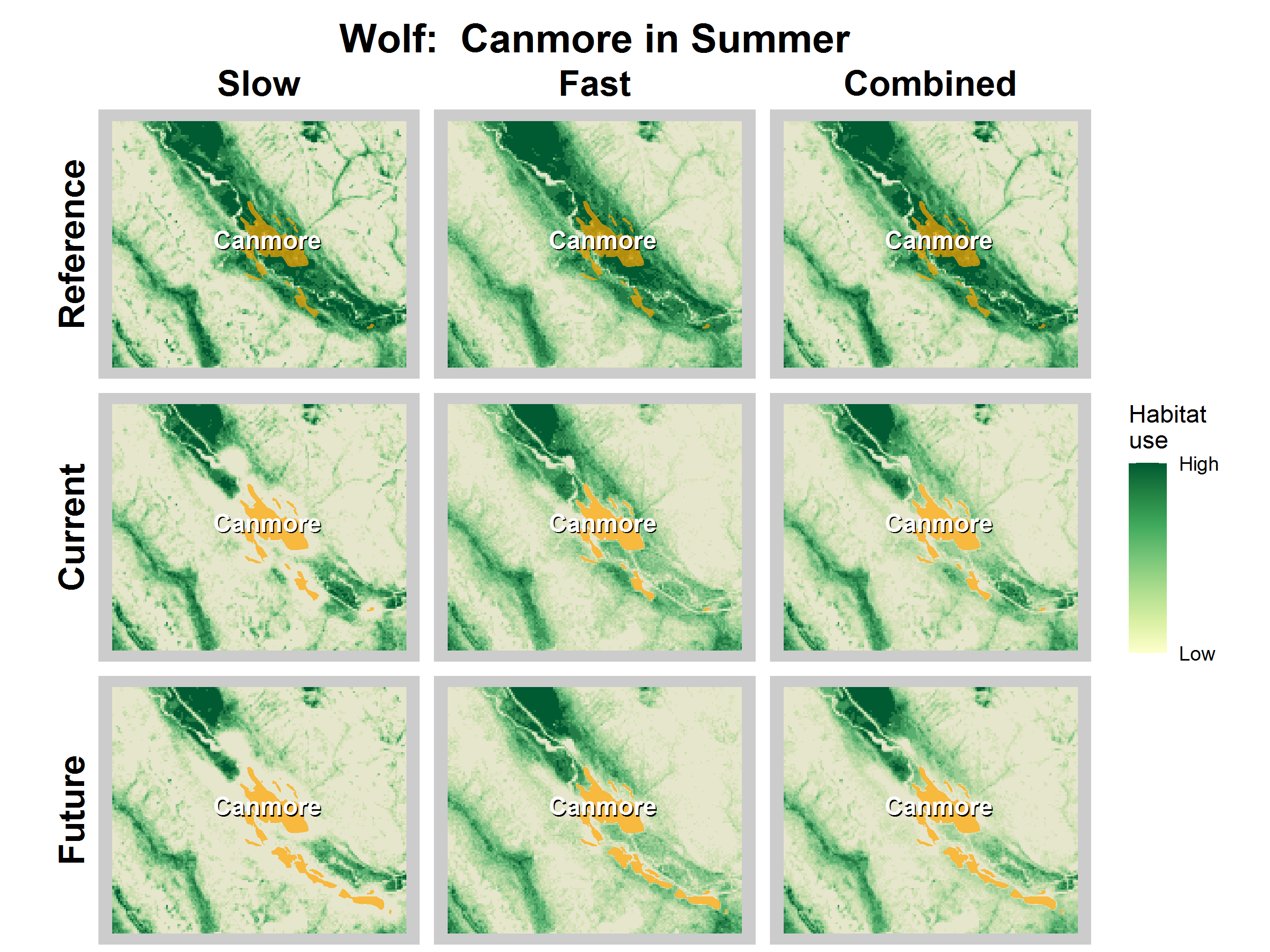


Figure S7. Wolf predicted habitat use by movement state and time period around Canmore, Alberta.
