# Supplementary material for: Towns and trails drive carnivore movement behaviour, resource selection, and connectivity
Source: Mov Ecol. 2022 Apr 8;10:17. doi: 10.1186/s40462-022-00318-5 (PMC8994267; doi:10.1186/s40462-022-00318-5)
Supplement: Supplementary file 2 — Additional file 2. Parameter estimates from hidden Markov movement models and step selection functions. [file 40462_2022_318_MOESM2_ESM.docx]

**Towns and Trails Drive Carnivore Movement Behaviour, Resource Selection, and Connectivity**

J. Whittington, M. Hebblewhite, R. Baron, A.T. Ford, J. Paczkowski

Movement Ecology

# Appendix S2: Tables of parameter estimates from movement models, step selection functions, and resource selection functions.

## Section 2.1 Explanatory variables

Table S1. Explanatory variables used and considered in wolf and grizzly bear step selection and resource selection analyses. We standardized most continuous variables by their mean and standard deviation to improve convergence and interpretability. Otherwise, we applied a decay function to most distance covariates so that the effect of the covariate declined with distance with an asymptote occurring around 500 m.

| Explanatory Variable | Transformation |
| --- | --- |
| Seasonal normalized difference vegetation index (NDVI) minus May NDVI | (x – mean(x)) / sd(x) |
| Seasonal fractional snow cover. For winter, average fractional snow cover. Linear &  quadratic | (x – mean(x)) / sd(x) |
| Slope (degrees) | (x – mean(x)) / sd(x) |
| Aspect SSW [-1 * cosine(aspect - 15)]; (1 = SSW, -1 = NNE, 0 = ENE or WSW) | (x – mean(x)) / sd(x) |
| Land Cover |  |
| Forest – closed coniferous (reference category*)* |  |
| Forest – deciduous and coniferous |  |
| Herbaceous |  |
| Shrub |  |
| Barren and rock |  |
| Distance to edge of forest (m) | 1 – exp(-10 * x * 0.001) |
| Distance (m) to large vegetated patch > 9 km^2^ | (x – mean(x)) / sd(x) |
| Burned (0 = no, 1 = forest burned since 1960) |  |
| Proximity to town (m) | exp(-1 * x * 0.001)  exp(-5 * x * 0.001)  exp(-10 * x * 0.001)  if x < 500, then x / 500, otherwise, 1.0  if x < 5000, then x / 5000, otherwise, 1.0 |
| Trail road density (km km^-2^) formal trails, 500 m radius. Linear & quadratic | (x – 1) / 2  (x^2^ – 1) / 4 |
| Trail road density (km km^-2^) x ln(Distance to paved road (km)) |  |
| Off/on railway (0 = off, 1 = on) |  |
| Off/on road (0 = off, 1 = on) |  |
| Off/on trail (0 = off, 1 = on) |  |
| Seasonal normalized difference vegetation index (NDVI) minus May NDVI | (x – mean(x)) / sd(x) |
| Seasonal fractional snow cover. For winter, average fractional snow cover. Linear &  quadratic | (x – mean(x)) / sd(x) |
| Slope (degrees) | (x – mean(x)) / sd(x) |
| Aspect SSW [-1 * cosine(aspect - 15)]; (1 = SSW, -1 = NNE, 0 = ENE or WSW) | (x – mean(x)) / sd(x) |
| Land Cover |  |
| Forest – closed coniferous (reference category*)* |  |
| Forest – deciduous and coniferous |  |
| Herbaceous |  |
| Shrub |  |
| Barren and rock |  |
| Distance to edge of forest (m) | 1 – exp(-10 * x * 0.001) |
| Distance (m) to large vegetated patch > 9 km^2^ | (x – mean(x)) / sd(x) |
| Burned (0 = no, 1 = forest burned since 1960) |  |

## Section 2.2 Hidden Markov movement model results

Table S2. Parameter estimates from hidden Markov movement models (HMM). The movement models contained two movement states classified as slow (encamped) and fast (travelling and exploratory) and state specific parameters for step length (gamma distribution) and turn angle (von Mises distribution). The gamma distribution contained the parameters mean and standard deviation (SD). The link to from mean and SD to commonly used shape and rate is given by: shape = mean^2^/SD^2^ and rate = mean/SD^2^. The von Mises distribution for turn angles included mean and concentration. Movement state transitions depended on proximity to roads (linear decay to 5 km), trail and road density (km/km^2^, 500 m radius), and time of day such that Cosine Hour = cosine(Hour * π/12).

| *HMM*  *Species* | *Season* | *Type* | *Parameter* | *State* | *Estimate* | *LCL* | *UCL* |
| --- | --- | --- | --- | --- | --- | --- | --- |
| Grizzly Bear | Fall | Movement Parameter | Step Length Mean | Slow | 0.012 | 0.011 | 0.011 |
|  |  | Movement Parameter | Step Length Mean | Fast | 0.596 | 0.583 | 0.663 |
|  |  | Movement Parameter | Step Length SD | Slow | 0.010 | 0.010 | 0.011 |
|  |  | Movement Parameter | Step Length SD | Fast | 0.648 | 0.634 | 0.663 |
|  |  | Movement Parameter | Turn Angle Mean | Slow | 3.124 | 3.063 | 0.708 |
|  |  | Movement Parameter | Turn Angle Mean | Fast | -0.017 | -0.069 | 0.538 |
|  |  | Movement Parameter | Turn Angle Concentration | Slow | 0.663 | 0.619 | 0.708 |
|  |  | Movement Parameter | Turn Angle Concentration | Fast | 0.510 | 0.483 | 0.538 |
|  |  | Transition Coefficient | Intercept | Slow to Fast | 0.010 | -0.427 | 0.447 |
|  |  | Transition Coefficient | CosineNight | Slow to Fast | -0.765 | -0.874 | -0.656 |
|  |  | Transition Coefficient | DistancetoTown (5km) | Slow to Fast | -0.618 | -1.077 | -0.158 |
|  |  | Transition Coefficient | TrailRoad Density | Slow to Fast | 0.019 | -0.090 | 0.127 |
|  |  | Transition Coefficient | Intercept | Fast to Slow | -3.599 | -4.129 | -3.070 |
|  |  | Transition Coefficient | CosineNight | Fast to Slow | 3.992 | 3.743 | 4.241 |
|  |  | Transition Coefficient | DistancetoTown (5km) | Fast to Slow | 0.907 | 0.374 | 1.440 |
|  |  | Transition Coefficient | TrailRoad Density | Fast to Slow | -0.270 | -0.394 | -0.147 |
| Grizzly Bear | Spring | Movement Parameter | Step Length Mean | Slow | 0.101 | 0.095 | 0.133 |
|  |  | Movement Parameter | Step Length Mean | Fast | 1.091 | 1.048 | 1.040 |
|  |  | Movement Parameter | Step Length SD | Slow | 0.125 | 0.117 | 0.133 |
|  |  | Movement Parameter | Step Length SD | Fast | 1.012 | 0.984 | 1.040 |
|  |  | Movement Parameter | Turn Angle Mean | Slow | 3.103 | 3.022 | 0.386 |
|  |  | Movement Parameter | Turn Angle Mean | Fast | -0.003 | -0.049 | 0.829 |
|  |  | Movement Parameter | Turn Angle Concentration | Slow | 0.351 | 0.317 | 0.386 |
|  |  | Movement Parameter | Turn Angle Concentration | Fast | 0.778 | 0.729 | 0.829 |
|  |  | Transition Coefficient | Intercept | Slow to Fast | -1.873 | -2.113 | -1.633 |
|  |  | Transition Coefficient | CosineNight | Slow to Fast | -0.167 | -0.254 | -0.079 |
|  |  | Transition Coefficient | DistancetoTown (5km) | Slow to Fast | 0.300 | 0.043 | 0.556 |
|  |  | Transition Coefficient | TrailRoad Density | Slow to Fast | 0.090 | 0.028 | 0.152 |
|  |  | Transition Coefficient | Intercept | Fast to Slow | -0.790 | -1.077 | -0.504 |
|  |  | Transition Coefficient | CosineNight | Fast to Slow | 1.013 | 0.878 | 1.147 |
|  |  | Transition Coefficient | DistancetoTown (5km) | Fast to Slow | -0.414 | -0.710 | -0.118 |
|  |  | Transition Coefficient | TrailRoad Density | Fast to Slow | -0.088 | -0.166 | -0.009 |
| Grizzly Bear | Summer | Movement Parameter | Step Length Mean | Slow | 0.013 | 0.013 | 0.012 |
|  |  | Movement Parameter | Step Length Mean | Fast | 0.722 | 0.712 | 0.793 |
|  |  | Movement Parameter | Step Length SD | Slow | 0.011 | 0.011 | 0.012 |
|  |  | Movement Parameter | Step Length SD | Fast | 0.779 | 0.766 | 0.793 |
|  |  | Movement Parameter | Turn Angle Mean | Slow | 3.141 | 3.057 | 0.575 |
|  |  | Movement Parameter | Turn Angle Mean | Fast | -0.013 | -0.056 | 0.486 |
|  |  | Movement Parameter | Turn Angle Concentration | Slow | 0.529 | 0.483 | 0.575 |
|  |  | Movement Parameter | Turn Angle Concentration | Fast | 0.465 | 0.445 | 0.486 |
|  |  | Transition Coefficient | Intercept | Slow to Fast | 11.893 | 10.597 | 13.190 |
|  |  | Transition Coefficient | CosineNight | Slow to Fast | -14.322 | -15.646 | -12.998 |
|  |  | Transition Coefficient | DistancetoTown (5km) | Slow to Fast | -0.892 | -1.579 | -0.206 |
|  |  | Transition Coefficient | TrailRoad Density | Slow to Fast | 0.246 | 0.100 | 0.393 |
|  |  | Transition Coefficient | Intercept | Fast to Slow | -9.581 | -10.293 | -8.869 |
|  |  | Transition Coefficient | CosineNight | Fast to Slow | 9.547 | 8.905 | 10.188 |
|  |  | Transition Coefficient | DistancetoTown (5km) | Fast to Slow | 1.688 | 1.258 | 2.117 |
|  |  | Transition Coefficient | TrailRoad Density | Fast to Slow | -0.035 | -0.130 | 0.059 |
| Wolf | Fall | Movement Parameter | Step Length Mean | Slow | 0.026 | 0.024 | 0.029 |
|  |  | Movement Parameter | Step Length Mean | Fast | 1.968 | 1.899 | 2.330 |
|  |  | Movement Parameter | Step Length SD | Slow | 0.026 | 0.024 | 0.029 |
|  |  | Movement Parameter | Step Length SD | Fast | 2.251 | 2.176 | 2.330 |
|  |  | Movement Parameter | Turn Angle Mean | Slow | -3.109 | -3.223 | 0.459 |
|  |  | Movement Parameter | Turn Angle Mean | Fast | 0.032 | -0.101 | 0.329 |
|  |  | Movement Parameter | Turn Angle Concentration | Slow | 0.409 | 0.360 | 0.459 |
|  |  | Movement Parameter | Turn Angle Concentration | Fast | 0.289 | 0.249 | 0.329 |
|  |  | Transition Coefficient | Intercept | Slow to Fast | -0.698 | -1.139 | -0.258 |
|  |  | Transition Coefficient | CosineNight | Slow to Fast | -0.293 | -0.401 | -0.185 |
|  |  | Transition Coefficient | DistancetoTown (5km) | Slow to Fast | 0.039 | -0.417 | 0.495 |
|  |  | Transition Coefficient | TrailRoad Density | Slow to Fast | -0.009 | -0.143 | 0.125 |
|  |  | Transition Coefficient | Intercept | Fast to Slow | -1.411 | -1.837 | -0.984 |
|  |  | Transition Coefficient | CosineNight | Fast to Slow | -0.080 | -0.184 | 0.025 |
|  |  | Transition Coefficient | DistancetoTown (5km) | Fast to Slow | 0.230 | -0.208 | 0.668 |
|  |  | Transition Coefficient | TrailRoad Density | Fast to Slow | -0.177 | -0.296 | -0.058 |
| Wolf | Spring | Movement Parameter | Step Length Mean | Slow | 0.034 | 0.032 | 0.034 |
|  |  | Movement Parameter | Step Length Mean | Fast | 2.068 | 2.008 | 2.478 |
|  |  | Movement Parameter | Step Length SD | Slow | 0.032 | 0.031 | 0.034 |
|  |  | Movement Parameter | Step Length SD | Fast | 2.407 | 2.338 | 2.478 |
|  |  | Movement Parameter | Turn Angle Mean | Slow | 3.066 | 2.995 | 0.565 |
|  |  | Movement Parameter | Turn Angle Mean | Fast | -0.017 | -0.110 | 0.383 |
|  |  | Movement Parameter | Turn Angle Concentration | Slow | 0.524 | 0.483 | 0.565 |
|  |  | Movement Parameter | Turn Angle Concentration | Fast | 0.349 | 0.316 | 0.383 |
|  |  | Transition Coefficient | Intercept | Slow to Fast | 0.417 | -0.100 | 0.934 |
|  |  | Transition Coefficient | CosineNight | Slow to Fast | 0.200 | 0.112 | 0.288 |
|  |  | Transition Coefficient | DistancetoTown (5km) | Slow to Fast | -1.302 | -1.826 | -0.777 |
|  |  | Transition Coefficient | TrailRoad Density | Slow to Fast | -0.471 | -0.603 | -0.340 |
|  |  | Transition Coefficient | Intercept | Fast to Slow | -1.608 | -2.062 | -1.155 |
|  |  | Transition Coefficient | CosineNight | Fast to Slow | -0.286 | -0.377 | -0.196 |
|  |  | Transition Coefficient | DistancetoTown (5km) | Fast to Slow | 0.393 | -0.070 | 0.856 |
|  |  | Transition Coefficient | TrailRoad Density | Fast to Slow | -0.121 | -0.227 | -0.015 |
| Wolf | Summer | Movement Parameter | Step Length Mean | Slow | 0.036 | 0.034 | 0.042 |
|  |  | Movement Parameter | Step Length Mean | Fast | 1.977 | 1.917 | 2.312 |
|  |  | Movement Parameter | Step Length SD | Slow | 0.039 | 0.035 | 0.042 |
|  |  | Movement Parameter | Step Length SD | Fast | 2.250 | 2.188 | 2.312 |
|  |  | Movement Parameter | Turn Angle Mean | Slow | 3.103 | 3.009 | 0.486 |
|  |  | Movement Parameter | Turn Angle Mean | Fast | 0.054 | -0.045 | 0.347 |
|  |  | Movement Parameter | Turn Angle Concentration | Slow | 0.440 | 0.394 | 0.486 |
|  |  | Movement Parameter | Turn Angle Concentration | Fast | 0.314 | 0.282 | 0.347 |
|  |  | Transition Coefficient | Intercept | Slow to Fast | -1.000 | -1.652 | -0.348 |
|  |  | Transition Coefficient | CosineNight | Slow to Fast | -0.050 | -0.143 | 0.044 |
|  |  | Transition Coefficient | DistancetoTown (5km) | Slow to Fast | 0.496 | -0.172 | 1.164 |
|  |  | Transition Coefficient | TrailRoad Density | Slow to Fast | -0.023 | -0.153 | 0.108 |
|  |  | Transition Coefficient | Intercept | Fast to Slow | -1.518 | -2.125 | -0.911 |
|  |  | Transition Coefficient | CosineNight | Fast to Slow | -0.143 | -0.232 | -0.054 |
|  |  | Transition Coefficient | DistancetoTown (5km) | Fast to Slow | 0.251 | -0.369 | 0.870 |
|  |  | Transition Coefficient | TrailRoad Density | Fast to Slow | -0.235 | -0.350 | -0.121 |
| Wolf | Winter | Movement Parameter | Step Length Mean | Slow | 0.019 | 0.018 | 0.020 |
|  |  | Movement Parameter | Step Length Mean | Fast | 1.602 | 1.551 | 1.969 |
|  |  | Movement Parameter | Step Length SD | Slow | 0.018 | 0.017 | 0.020 |
|  |  | Movement Parameter | Step Length SD | Fast | 1.908 | 1.849 | 1.969 |
|  |  | Movement Parameter | Turn Angle Mean | Slow | 3.033 | 2.955 | 0.607 |
|  |  | Movement Parameter | Turn Angle Mean | Fast | -0.053 | -0.140 | 0.435 |
|  |  | Movement Parameter | Turn Angle Concentration | Slow | 0.559 | 0.511 | 0.607 |
|  |  | Movement Parameter | Turn Angle Concentration | Fast | 0.398 | 0.361 | 0.435 |
|  |  | Transition Coefficient | Intercept | Slow to Fast | 0.262 | -0.169 | 0.694 |
|  |  | Transition Coefficient | CosineNight | Slow to Fast | -0.420 | -0.521 | -0.319 |
|  |  | Transition Coefficient | DistancetoTown (5km) | Slow to Fast | -0.979 | -1.428 | -0.530 |
|  |  | Transition Coefficient | TrailRoad Density | Slow to Fast | -0.005 | -0.125 | 0.116 |
|  |  | Transition Coefficient | Intercept | Fast to Slow | -1.321 | -1.676 | -0.967 |
|  |  | Transition Coefficient | CosineNight | Fast to Slow | -0.166 | -0.262 | -0.070 |
|  |  | Transition Coefficient | DistancetoTown (5km) | Fast to Slow | 0.076 | -0.293 | 0.445 |
|  |  | Transition Coefficient | TrailRoad Density | Fast to Slow | -0.290 | -0.398 | -0.182 |

## Section 2.3 Step selection function results

Table S3. Step selection function parameter estimates and 95% confidence intervals for grizzly bears and wolves by season in Banff National Park and surrounding areas, 2002 - 2020.

| *SSF*  *Species* | *Season* | *Parameter* | *Estimate* | *SE* | *Statistic* | *p* | *LCL* | *UCL* |
| --- | --- | --- | --- | --- | --- | --- | --- | --- |
| Grizzly Bear | Fall | Burned since 1960 | 0.36 | 0.07 | 2.2 | 0.030 | 0.04 | 0.69 |
|  |  | Cosine Turn Angle | 0.87 | 0.04 | 13.3 | <0.001 | 0.74 | 0.99 |
|  |  | Cosine Turn Angle:FastState | -0.11 | 0.06 | -1.4 | 0.155 | -0.26 | 0.04 |
|  |  | Distance to Forest Edge | -0.25 | 0.06 | -2.3 | 0.021 | -0.46 | -0.04 |
|  |  | Distance to Patch > 9 km2 | -0.55 | 0.06 | -4.3 | <0.001 | -0.81 | -0.30 |
|  |  | Fast | -6.49 | 0.10 | -15.6 | <0.001 | -7.31 | -5.68 |
|  |  | Landcover: Herbaceous | 0.23 | 0.05 | 2.5 | 0.012 | 0.05 | 0.40 |
|  |  | Landcover: Open Conifer or Deciduous | 0.16 | 0.04 | 3.1 | 0.002 | 0.06 | 0.26 |
|  |  | Landcover: Shrub | 0.07 | 0.05 | 0.7 | 0.460 | -0.12 | 0.26 |
|  |  | logStepLength | -1.51 | 0.02 | -20.2 | <0.001 | -1.66 | -1.37 |
|  |  | NDVI | 0.35 | 0.02 | 7.4 | <0.001 | 0.26 | 0.44 |
|  |  | Near Town | -2.79 | 0.79 | -5.8 | <0.001 | -3.73 | -1.86 |
|  |  | Near Town:Fast | 2.45 | 0.82 | 4.6 | <0.001 | 1.40 | 3.50 |
|  |  | Railway | -5.40 | 1.13 | -2.4 | 0.015 | -9.74 | -1.06 |
|  |  | Railway:Fast | 7.16 | 1.22 | 3.0 | 0.003 | 2.51 | 11.82 |
|  |  | Road | -5.47 | 1.86 | -1.5 | 0.125 | -12.47 | 1.52 |
|  |  | Road:Fast | 5.30 | 2.00 | 1.4 | 0.171 | -2.29 | 12.88 |
|  |  | Slope | 0.13 | 0.03 | 2.2 | 0.029 | 0.01 | 0.25 |
|  |  | SnowCover | 0.50 | 0.16 | 1.7 | 0.084 | -0.07 | 1.07 |
|  |  | SnowCover - Quadratic | -0.72 | 0.27 | -1.6 | 0.108 | -1.61 | 0.16 |
|  |  | SSW Aspect | -0.04 | 0.03 | -0.7 | 0.470 | -0.16 | 0.07 |
|  |  | Step Length:Fast | 2.33 | 0.02 | 32.7 | <0.001 | 2.19 | 2.47 |
|  |  | Trail | -2.39 | 0.45 | -3.0 | 0.003 | -3.96 | -0.82 |
|  |  | Trail:Fast | 2.80 | 0.51 | 2.9 | 0.003 | 0.93 | 4.67 |
|  |  | TrailRoadDensity | 0.03 | 0.05 | 0.2 | 0.868 | -0.28 | 0.34 |
|  |  | TrailRoadDensity - Quadratic | 0.02 | 0.01 | 1.6 | 0.109 | -0.00 | 0.05 |
|  |  | TrailRoadDensity:Night | 0.08 | 0.02 | 3.1 | 0.002 | 0.03 | 0.13 |
| Grizzly Bear | Spring | Burned since 1960 | 0.15 | 0.06 | 1.1 | 0.271 | -0.11 | 0.41 |
|  |  | Cosine Turn Angle | 0.83 | 0.03 | 39.1 | <0.001 | 0.79 | 0.87 |
|  |  | Cosine Turn Angle:FastState | 0.26 | 0.05 | 5.6 | <0.001 | 0.17 | 0.34 |
|  |  | Distance to Forest Edge | -0.96 | 0.05 | -11.2 | <0.001 | -1.13 | -0.79 |
|  |  | Distance to Patch > 9 km2 | -0.50 | 0.07 | -2.0 | 0.044 | -0.98 | -0.01 |
|  |  | Fast | -14.22 | 0.14 | -22.0 | <0.001 | -15.48 | -12.96 |
|  |  | Landcover: Barren | 0.23 | 0.04 | 2.7 | 0.007 | 0.06 | 0.40 |
|  |  | Landcover: Herbaceous | 0.47 | 0.05 | 4.5 | <0.001 | 0.26 | 0.67 |
|  |  | Landcover: Open Conifer or Deciduous | 0.20 | 0.03 | 4.4 | <0.001 | 0.11 | 0.28 |
|  |  | Landcover: Shrub | 0.39 | 0.04 | 7.1 | <0.001 | 0.28 | 0.50 |
|  |  | logStepLength | -0.67 | 0.01 | -32.6 | <0.001 | -0.71 | -0.63 |
|  |  | NDVI | 0.37 | 0.03 | 8.2 | <0.001 | 0.28 | 0.46 |
|  |  | Near Town | -0.66 | 0.16 | -3.0 | 0.003 | -1.10 | -0.23 |
|  |  | Railway | -0.92 | 0.17 | -2.8 | 0.005 | -1.58 | -0.27 |
|  |  | Railway:Fast | 3.22 | 0.25 | 7.3 | <0.001 | 2.35 | 4.09 |
|  |  | Road | -1.41 | 0.15 | -5.6 | <0.001 | -1.90 | -0.91 |
|  |  | Road:Fast | 1.91 | 0.22 | 7.6 | <0.001 | 1.42 | 2.40 |
|  |  | Slope | -0.33 | 0.05 | -3.4 | 0.001 | -0.52 | -0.14 |
|  |  | Slope -Quadratic | 0.30 | 0.07 | 3.0 | 0.003 | 0.10 | 0.49 |
|  |  | SnowCover | -0.21 | 0.05 | -1.9 | 0.059 | -0.43 | 0.01 |
|  |  | SnowCover - Quadratic | 0.07 | 0.04 | 1.0 | 0.323 | -0.07 | 0.22 |
|  |  | SSW Aspect | -0.13 | 0.02 | -2.7 | 0.008 | -0.22 | -0.03 |
|  |  | Step Length:Fast | 2.28 | 0.02 | 34.3 | <0.001 | 2.15 | 2.41 |
|  |  | Trail | -0.76 | 0.14 | -2.5 | 0.014 | -1.37 | -0.15 |
|  |  | Trail:Fast | 1.25 | 0.21 | 2.7 | 0.006 | 0.35 | 2.14 |
|  |  | TrailDensity:logDistPavedRoad | -0.23 | 0.10 | -1.5 | 0.143 | -0.54 | 0.08 |
|  |  | TrailRoadDensity | 0.12 | 0.03 | 1.4 | 0.151 | -0.04 | 0.29 |
|  |  | TrailRoadDensity - Quadratic | -0.01 | 0.00 | -1.1 | 0.251 | -0.04 | 0.01 |
|  |  | TrailRoadDensity:Fast | 0.05 | 0.03 | 0.6 | 0.557 | -0.12 | 0.21 |
|  |  | TrailRoadDensity:Night | 0.03 | 0.01 | 1.3 | 0.200 | -0.02 | 0.08 |
| Grizzly Bear | Summer | Cosine Turn Angle | 0.87 | 0.05 | 8.7 | <0.001 | 0.67 | 1.06 |
|  |  | Cosine Turn Angle:FastState | -0.26 | 0.06 | -2.3 | 0.021 | -0.48 | -0.04 |
|  |  | Distance to Forest Edge | -0.79 | 0.05 | -5.9 | <0.001 | -1.05 | -0.53 |
|  |  | Distance to Patch > 9 km2 | -0.60 | 0.07 | -5.2 | <0.001 | -0.82 | -0.37 |
|  |  | Fast | -5.28 | 0.09 | -17.9 | <0.001 | -5.85 | -4.70 |
|  |  | Landcover: Barren | -0.14 | 0.05 | -1.6 | 0.113 | -0.32 | 0.03 |
|  |  | Landcover: Herbaceous | 0.14 | 0.05 | 1.1 | 0.255 | -0.10 | 0.39 |
|  |  | Landcover: Open Conifer or Deciduous | 0.18 | 0.03 | 2.7 | 0.007 | 0.05 | 0.31 |
|  |  | Landcover: Shrub | 0.14 | 0.04 | 1.4 | 0.162 | -0.06 | 0.33 |
|  |  | logStepLength | -2.12 | 0.02 | -41.7 | <0.001 | -2.22 | -2.02 |
|  |  | NDVI | 0.30 | 0.02 | 10.5 | <0.001 | 0.25 | 0.36 |
|  |  | Near Town | -0.38 | 0.20 | -1.2 | 0.226 | -1.00 | 0.24 |
|  |  | Near Town:Night | 0.45 | 0.23 | 1.8 | 0.068 | -0.03 | 0.94 |
|  |  | Road | -3.89 | 0.68 | -2.2 | 0.026 | -7.31 | -0.48 |
|  |  | Road:Fast | 4.43 | 0.73 | 2.4 | 0.019 | 0.74 | 8.13 |
|  |  | Slope -Quadratic | -0.21 | 0.03 | -2.6 | 0.010 | -0.37 | -0.05 |
|  |  | SnowCover | -1.64 | 0.29 | -3.1 | 0.002 | -2.69 | -0.60 |
|  |  | Step Length:Fast | 2.72 | 0.02 | 45.2 | <0.001 | 2.60 | 2.83 |
|  |  | Trail | -2.37 | 0.38 | -3.4 | 0.001 | -3.74 | -1.01 |
|  |  | Trail:Fast | 2.66 | 0.41 | 3.6 | <0.001 | 1.21 | 4.11 |
|  |  | TrailDensity:logDistPavedRoad | 0.28 | 0.07 | 1.7 | 0.087 | -0.04 | 0.60 |
|  |  | TrailRoadDensity | 0.03 | 0.05 | 0.3 | 0.773 | -0.18 | 0.25 |
|  |  | TrailRoadDensity - Quadratic | -0.01 | 0.00 | -1.8 | 0.080 | -0.02 | 0.00 |
|  |  | TrailRoadDensity:Fast | 0.09 | 0.04 | 1.3 | 0.200 | -0.05 | 0.24 |
|  |  | TrailRoadDensity:Night | 0.06 | 0.01 | 2.4 | 0.015 | 0.01 | 0.10 |
| Wolf | Fall | Cosine Turn Angle | 1.13 | 0.04 | 19.9 | <0.001 | 1.01 | 1.24 |
|  |  | Cosine Turn Angle:FastState | -0.25 | 0.06 | -2.6 | 0.009 | -0.44 | -0.06 |
|  |  | Distance to Forest Edge | -0.23 | 0.07 | -2.3 | 0.023 | -0.42 | -0.03 |
|  |  | Distance to Patch > 9 km2 | -0.68 | 0.13 | -2.6 | 0.009 | -1.19 | -0.17 |
|  |  | Fast | -9.01 | 0.13 | -13.8 | <0.001 | -10.29 | -7.73 |
|  |  | Landcover: Barren | -0.13 | 0.08 | -1.5 | 0.140 | -0.31 | 0.04 |
|  |  | Landcover: Herbaceous | 0.21 | 0.06 | 3.6 | <0.001 | 0.10 | 0.33 |
|  |  | Landcover: Open Conifer or Deciduous | 0.08 | 0.04 | 2.2 | 0.025 | 0.01 | 0.15 |
|  |  | Landcover: Shrub | 0.25 | 0.06 | 2.8 | 0.005 | 0.07 | 0.42 |
|  |  | logStepLength | -0.99 | 0.01 | -37.4 | <0.001 | -1.04 | -0.94 |
|  |  | NDVI | 0.39 | 0.03 | 6.2 | <0.001 | 0.27 | 0.51 |
|  |  | Near Town | -0.75 | 0.43 | -0.8 | 0.428 | -2.59 | 1.10 |
|  |  | Road | -5.55 | 1.75 | -1.5 | 0.145 | -13.01 | 1.92 |
|  |  | Road:Fast | 6.32 | 1.91 | 1.6 | 0.119 | -1.62 | 14.26 |
|  |  | Slope | -0.59 | 0.09 | -3.3 | 0.001 | -0.94 | -0.24 |
|  |  | Slope -Quadratic | -0.25 | 0.15 | -0.9 | 0.353 | -0.76 | 0.27 |
|  |  | SnowCover | -1.24 | 0.17 | -6.1 | <0.001 | -1.63 | -0.84 |
|  |  | SnowCover - Quadratic | 1.12 | 0.30 | 4.7 | <0.001 | 0.65 | 1.59 |
|  |  | Step Length:Fast | 1.99 | 0.02 | 25.9 | <0.001 | 1.84 | 2.14 |
|  |  | Trail | -1.51 | 0.24 | -3.8 | <0.001 | -2.28 | -0.74 |
|  |  | Trail:Fast | 2.21 | 0.31 | 4.5 | <0.001 | 1.24 | 3.17 |
|  |  | TrailDensity:logDistPavedRoad | 0.30 | 0.07 | 1.0 | 0.298 | -0.27 | 0.87 |
|  |  | TrailRoadDensity | -0.30 | 0.08 | -1.2 | 0.239 | -0.79 | 0.20 |
|  |  | TrailRoadDensity - Quadratic | -0.07 | 0.01 | -1.5 | 0.129 | -0.15 | 0.02 |
|  |  | TrailRoadDensity:Fast | 0.46 | 0.07 | 3.7 | <0.001 | 0.22 | 0.70 |
|  |  | TrailRoadDensity:Night | 0.27 | 0.03 | 3.4 | 0.001 | 0.11 | 0.43 |
| Wolf | Spring | Cosine Turn Angle | 1.14 | 0.03 | 22.3 | <0.001 | 1.04 | 1.24 |
|  |  | Cosine Turn Angle:FastState | -0.17 | 0.05 | -2.1 | 0.033 | -0.32 | -0.01 |
|  |  | Distance to Forest Edge | -0.39 | 0.05 | -3.4 | 0.001 | -0.62 | -0.17 |
|  |  | Distance to Patch > 9 km2 | -0.77 | 0.11 | -3.8 | <0.001 | -1.17 | -0.37 |
|  |  | Fast | -9.53 | 0.11 | -16.2 | <0.001 | -10.68 | -8.38 |
|  |  | Landcover: Barren | -0.20 | 0.06 | -2.1 | 0.040 | -0.40 | -0.01 |
|  |  | Landcover: Herbaceous | 0.13 | 0.05 | 1.2 | 0.218 | -0.08 | 0.33 |
|  |  | Landcover: Open Conifer or Deciduous | 0.05 | 0.03 | 0.9 | 0.386 | -0.06 | 0.16 |
|  |  | Landcover: Shrub | 0.39 | 0.04 | 3.5 | 0.001 | 0.17 | 0.61 |
|  |  | logStepLength | -0.89 | 0.01 | -28.2 | <0.001 | -0.95 | -0.83 |
|  |  | NDVI | 0.28 | 0.04 | 5.9 | <0.001 | 0.19 | 0.37 |
|  |  | Near Town | -12.09 | 3.06 | -2.3 | 0.021 | -22.37 | -1.82 |
|  |  | Near Town:Fast | 12.53 | 3.25 | 2.7 | 0.008 | 3.31 | 21.75 |
|  |  | Railway | -4.76 | 2.06 | -1.2 | 0.220 | -12.36 | 2.84 |
|  |  | Railway:Fast | 5.73 | 2.28 | 1.4 | 0.166 | -2.37 | 13.84 |
|  |  | Road | -7.06 | 1.92 | -1.0 | 0.303 | -20.48 | 6.37 |
|  |  | Road:Fast | 7.77 | 2.06 | 1.1 | 0.286 | -6.52 | 22.06 |
|  |  | Slope | -0.90 | 0.07 | -4.2 | <0.001 | -1.31 | -0.48 |
|  |  | Slope -Quadratic | 0.41 | 0.11 | 1.6 | 0.120 | -0.11 | 0.92 |
|  |  | SnowCover | -0.59 | 0.06 | -3.7 | <0.001 | -0.90 | -0.28 |
|  |  | SnowCover - Quadratic | 0.32 | 0.06 | 2.4 | 0.015 | 0.06 | 0.58 |
|  |  | SSW Aspect | 0.28 | 0.02 | 4.7 | <0.001 | 0.17 | 0.40 |
|  |  | Step Length:Fast | 1.93 | 0.02 | 28.3 | <0.001 | 1.79 | 2.06 |
|  |  | Trail | -0.56 | 0.13 | -1.3 | 0.193 | -1.40 | 0.28 |
|  |  | Trail:Fast | 0.96 | 0.19 | 1.6 | 0.106 | -0.20 | 2.13 |
|  |  | TrailDensity:logDistPavedRoad | 0.10 | 0.06 | 0.6 | 0.578 | -0.24 | 0.44 |
|  |  | TrailRoadDensity | 0.33 | 0.07 | 1.3 | 0.195 | -0.17 | 0.84 |
|  |  | TrailRoadDensity - Quadratic | -0.11 | 0.02 | -1.5 | 0.132 | -0.26 | 0.03 |
|  |  | TrailRoadDensity:Night | 0.29 | 0.03 | 5.3 | <0.001 | 0.18 | 0.40 |
| Wolf | Summer | Burned since 1960 | 0.23 | 0.04 | 2.4 | 0.017 | 0.04 | 0.42 |
|  |  | Cosine Turn Angle | 1.05 | 0.04 | 18.5 | <0.001 | 0.94 | 1.16 |
|  |  | Cosine Turn Angle:FastState | -0.19 | 0.05 | -2.5 | 0.012 | -0.34 | -0.04 |
|  |  | Distance to Patch > 9 km2 | -0.46 | 0.08 | -3.4 | 0.001 | -0.72 | -0.19 |
|  |  | Fast | -8.24 | 0.11 | -21.4 | <0.001 | -9.00 | -7.49 |
|  |  | Landcover: Barren | -0.21 | 0.05 | -2.1 | 0.032 | -0.40 | -0.02 |
|  |  | Landcover: Herbaceous | -0.14 | 0.04 | -1.2 | 0.244 | -0.38 | 0.10 |
|  |  | logStepLength | -0.98 | 0.01 | -33.6 | <0.001 | -1.03 | -0.92 |
|  |  | NDVI | 0.31 | 0.02 | 6.1 | <0.001 | 0.21 | 0.41 |
|  |  | Near Town | -25.42 | 7.75 | -3.7 | <0.001 | -38.77 | -12.06 |
|  |  | Near Town:Fast | 25.81 | 8.06 | 3.4 | 0.001 | 10.85 | 40.78 |
|  |  | Railway | -3.77 | 1.83 | -1.6 | 0.110 | -8.39 | 0.85 |
|  |  | Railway:Fast | 5.09 | 2.04 | 1.9 | 0.058 | -0.17 | 10.35 |
|  |  | Slope | -0.89 | 0.03 | -12.8 | <0.001 | -1.02 | -0.75 |
|  |  | SnowCover | -0.96 | 0.30 | -1.9 | 0.056 | -1.95 | 0.02 |
|  |  | SSW Aspect | 0.15 | 0.02 | 3.8 | <0.001 | 0.07 | 0.24 |
|  |  | Step Length:Fast | 1.89 | 0.02 | 36.2 | <0.001 | 1.78 | 1.99 |
|  |  | Trail | -2.92 | 0.40 | -4.7 | <0.001 | -4.15 | -1.69 |
|  |  | Trail:Fast | 3.72 | 0.46 | 5.2 | <0.001 | 2.31 | 5.13 |
|  |  | TrailDensity:logDistPavedRoad | -0.33 | 0.07 | -1.6 | 0.120 | -0.75 | 0.09 |
|  |  | TrailRoadDensity | 0.19 | 0.08 | 0.9 | 0.356 | -0.21 | 0.58 |
|  |  | TrailRoadDensity - Quadratic | -0.09 | 0.02 | -1.2 | 0.216 | -0.23 | 0.05 |
|  |  | TrailRoadDensity:Fast | 0.22 | 0.06 | 1.2 | 0.212 | -0.12 | 0.56 |
|  |  | TrailRoadDensity:Night | 0.29 | 0.03 | 3.6 | <0.001 | 0.13 | 0.44 |
| Wolf | Winter | Cosine Turn Angle | 0.98 | 0.02 | 27.8 | <0.001 | 0.91 | 1.04 |
|  |  | Distance to Forest Edge | -0.31 | 0.06 | -2.8 | 0.005 | -0.53 | -0.10 |
|  |  | Distance to Patch > 9 km2 | -1.39 | 0.15 | -10.5 | <0.001 | -1.65 | -1.13 |
|  |  | Fast | -8.42 | 0.10 | -13.9 | <0.001 | -9.60 | -7.23 |
|  |  | Landcover: Barren | -0.21 | 0.07 | -2.8 | 0.005 | -0.35 | -0.06 |
|  |  | Landcover: Herbaceous | 0.26 | 0.05 | 4.6 | <0.001 | 0.15 | 0.37 |
|  |  | Landcover: Open Conifer or Deciduous | 0.09 | 0.04 | 1.8 | 0.077 | -0.01 | 0.20 |
|  |  | Landcover: Shrub | 0.20 | 0.05 | 3.3 | 0.001 | 0.08 | 0.33 |
|  |  | logStepLength | -1.08 | 0.01 | -43.7 | <0.001 | -1.13 | -1.04 |
|  |  | NDVI | 0.23 | 0.04 | 5.1 | <0.001 | 0.14 | 0.32 |
|  |  | Near Town | -10.33 | 2.20 | -4.1 | <0.001 | -15.24 | -5.42 |
|  |  | Near Town:Fast | 9.81 | 2.27 | 3.0 | 0.002 | 3.49 | 16.14 |
|  |  | Near Town:Night | 1.45 | 0.67 | 3.0 | 0.002 | 0.51 | 2.38 |
|  |  | Railway | -4.22 | 1.05 | -2.8 | 0.005 | -7.18 | -1.26 |
|  |  | Railway:Fast | 6.05 | 1.18 | 3.5 | <0.001 | 2.65 | 9.46 |
|  |  | Road | -16.67 | 4.64 | -1.3 | 0.194 | -41.82 | 8.48 |
|  |  | Road:Fast | 17.62 | 4.79 | 1.3 | 0.178 | -8.00 | 43.24 |
|  |  | Slope | -0.70 | 0.07 | -5.8 | <0.001 | -0.93 | -0.46 |
|  |  | Slope -Quadratic | 0.41 | 0.12 | 2.4 | 0.017 | 0.07 | 0.75 |
|  |  | SnowCover - Quadratic | -0.71 | 0.06 | -6.8 | <0.001 | -0.92 | -0.51 |
|  |  | Step Length:Fast | 2.03 | 0.02 | 29.8 | <0.001 | 1.89 | 2.16 |
|  |  | Trail | -1.74 | 0.30 | -2.1 | 0.038 | -3.38 | -0.09 |
|  |  | Trail:Fast | 2.29 | 0.37 | 2.5 | 0.011 | 0.53 | 4.06 |
|  |  | TrailDensity:logDistPavedRoad | 0.15 | 0.07 | 1.2 | 0.240 | -0.10 | 0.40 |
|  |  | TrailRoadDensity | -0.29 | 0.07 | -2.4 | 0.014 | -0.52 | -0.06 |
|  |  | TrailRoadDensity - Quadratic | -0.07 | 0.01 | -2.2 | 0.025 | -0.14 | -0.01 |
|  |  | TrailRoadDensity:Fast | 0.52 | 0.06 | 3.8 | <0.001 | 0.26 | 0.79 |
|  |  | TrailRoadDensity:Night | 0.25 | 0.03 | 2.6 | 0.009 | 0.06 | 0.43 |


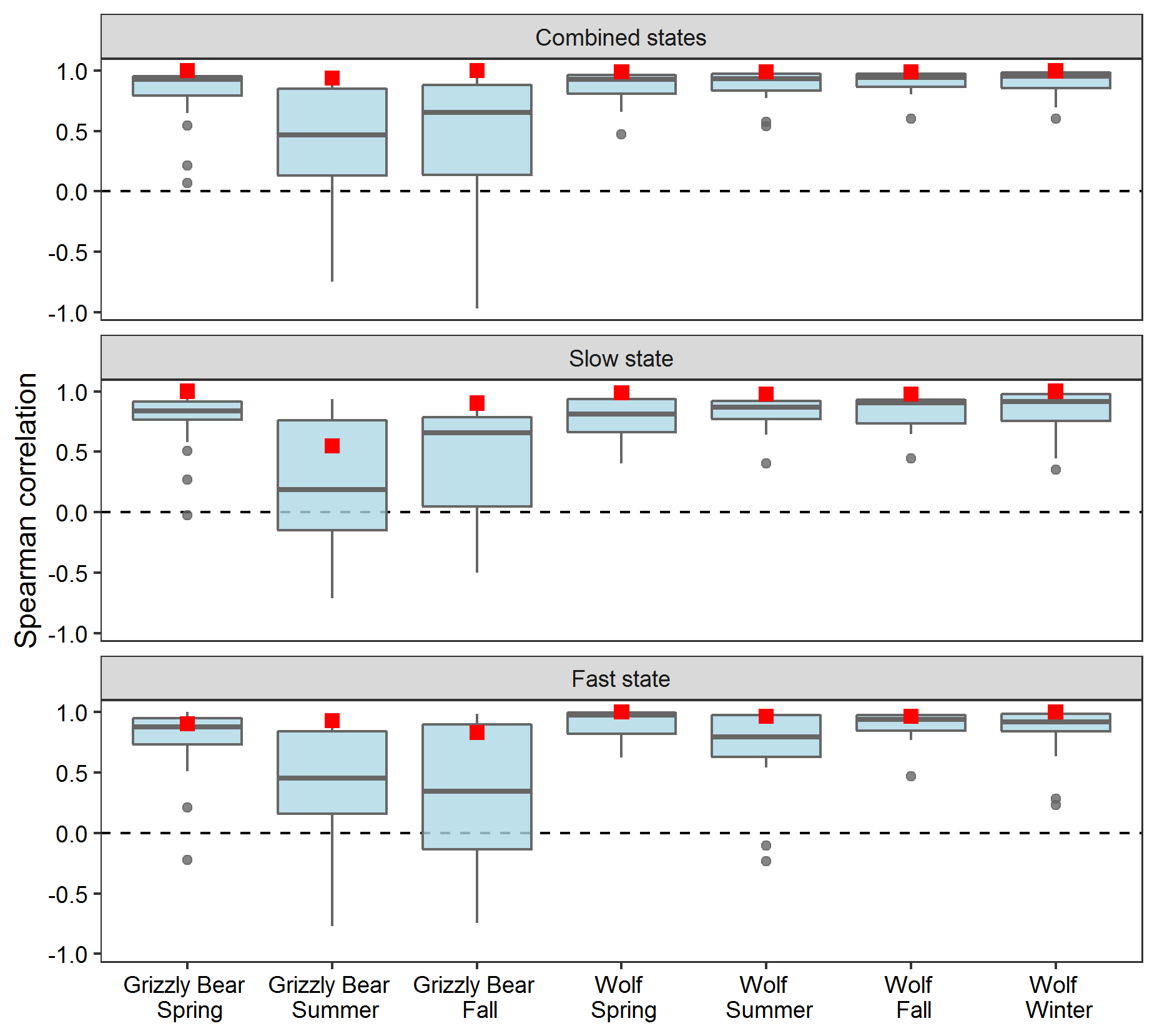


#### Figure S1. Model performance measured as the Spearman rank correlation coefficients between number of GPS locations versus bin rank from predictive maps showing intensity of use. Predictive maps were generated by simulating animal movements from movement models and step selection functions. Red squares indicate correlation coefficients for pooled animals and boxplots show variation in correlation coefficients for individual animals.

## Section 2.4 Slopes used by grizzly bears and wolves.

Table S4. Table of slopes used by grizzly bears and wolves in Banff National Park and surrounding areas, 2002 - 2020. Slopes (degrees) were calculated from a digital elevation model with a 30 m resolution. Values indicate percent of locations used by grizzly bears and wolves with values less than or equal to 25, 30, and 35 degrees respectively.

| Slope  cutoff | Grizzly bear | Wolf |
| --- | --- | --- |
| 25 | 77.7 | 95.4 |
| 30 | 88.5 | 98.1 |
| 35 | 96.1 | 99.5 |

## Section 2.5 Connectivity

Table S5. Merriam connectivity measured for as the number of simulated paths (NPaths) that travelled between patches or crossed digital transects relative to reference conditions (NReference) with no anthropogenic development. Patches were located 34 km apart west of Banff, AB near Vermilion Lakes and east of Canmore, AB within Bow Valley Provincial Park. Digital transect traversed the Bow Valley through Banff and Canmore. We simulated 200,000 paths within the 17,450 km^2^ study area for each species, season, and time period (landscape scenario).

| Type | Species | Season | TimePeriod | NPaths | NReference | Connectivity |
| --- | --- | --- | --- | --- | --- | --- |
| Patch | Grizzly Bear | Spring | Current | 2 | 9 | 0.22 |
|  | Grizzly Bear | Spring | Future | 1 | 9 | 0.11 |
|  | Grizzly Bear | Summer | Current | 0 | 8 | 0 |
|  | Grizzly Bear | Summer | Future | 0 | 8 | 0 |
|  | Wolf | Spring | Current | 100 | 657 | 0.15 |
|  | Wolf | Spring | Future | 73 | 657 | 0.11 |
|  | Wolf | Summer | Current | 49 | 327 | 0.15 |
|  | Wolf | Summer | Future | 50 | 327 | 0.15 |
|  | Wolf | Fall | Current | 25 | 352 | 0.07 |
|  | Wolf | Fall | Future | 23 | 352 | 0.07 |
|  | Wolf | Winter | Current | 36 | 393 | 0.09 |
|  | Wolf | Winter | Future | 24 | 393 | 0.06 |
| Transect | Grizzly Bear | Spring | Current | 2462 | 14325 | 0.17 |
|  | Grizzly Bear | Spring | Future | 2432 | 14325 | 0.17 |
|  | Grizzly Bear | Summer | Current | 1244 | 8219 | 0.15 |
|  | Grizzly Bear | Summer | Future | 1212 | 8219 | 0.15 |
|  | Grizzly Bear | Fall | Current | 721 | 3201 | 0.23 |
|  | Grizzly Bear | Fall | Future | 868 | 3201 | 0.27 |
|  | Wolf | Spring | Current | 5278 | 35838 | 0.15 |
|  | Wolf | Spring | Future | 3832 | 35838 | 0.11 |
|  | Wolf | Summer | Current | 3819 | 22000 | 0.17 |
|  | Wolf | Summer | Future | 3065 | 22000 | 0.14 |
|  | Wolf | Fall | Current | 3166 | 29316 | 0.11 |
|  | Wolf | Fall | Future | 2471 | 29316 | 0.08 |
|  | Wolf | Winter | Current | 2256 | 32380 | 0.07 |
|  | Wolf | Winter | Future | 1698 | 32380 | 0.05 |
